# A folate receptor 3 SNP promotes mitochondria‐induced clonogenicity of CML leukemia cells: Implications for treatment free remission

**DOI:** 10.1002/ctm2.317

**Published:** 2021-02-04

**Authors:** Na Shen, Teng Liu, Wen Liu, Zhaodong Zhong, Qing Li, Xiaoying Zhu, Ping Zou, Yong You, An‐Yuan Guo, Xiaojian Zhu

**Affiliations:** ^1^ Department of Hematology, Tongji Hospital, Tongji Medical College Huazhong University of Science and Technology Wuhan China; ^2^ Key Laboratory of Molecular Biophysics of the Ministry of Education, College of Life Science and Technology Huazhong University of Science and Technology Wuhan China; ^3^ Department of Hematology The First Affiliated Hospital of Zhengzhou University Zhengzhou China; ^4^ Institute of Hematology, Union Hospital, Tongji Medical College Huazhong University of Science and Technology Wuhan China; ^5^ Department of Hematology Wuhan No.1 Hospital Wuhan China

Dear Editor,

Treatment‐free remission (TFR) is an emerging goal of chronic myeloid leukemia (CML) due to long‐term costs and toxicity.^1^, ^2^ Previous studies had shown that 55% of selected CML patients suffered molecular relapse after tyrosine kinase inhibitor (TKI) cessation, while the rest remained in a TFR.^3^, ^4^ Here, we highlighted the biological role and indicator of folate receptor 3 (FOLR3) and its SNP in TFR.

We collected bone marrow samples from CML patients at the time of TKI cessation. These patients were followed up for 24 months; from them 7 relapsed and 7 non‐relapsed were sequenced (Table S1). Among top differentially expressed genes (DEGs) (Figure 1A), the FOLR3, which was not expressed in any relapsed samples and highly expressed in 3/7 non‐relapsed samples, was the most significantly gene (Fig S1B). Published expression profiles (Figure 1B) indicated FOLR3 highly expressed in bone marrow. We found that FOLR3 was highly expressed in TKI responders by analysing two public CML datasets, GSE14671^5^ and GSE2535^6^ (Figure 1C, D). Besides, we identified a TA insertion (SNP rs139130389, termed FOLR3 SNP+) in the third exon of FOLR3 gene in three non‐relapsed CML samples with FOLR3 overexpression (Figure 1E). The FOLR3 SNP+ encoded a functional protein; only a partial folate receptor domain was encoded by the FOLR3 SNP‐ (Figure 1E; Figure S1). Average proportion of FOLR3 SNP+ genotype in human is 11.08%, with Africans having the highest frequency (Figure 1F). We retrospectively studied 87 CML patients who discontinued TKI outside of clinical trials. TFR at 48 months for the FOLR3 SNP+ and FOLR3 SNP‐ patients were 84.4% (95% CI: 74.2%‐94.6%) and 52.4% (95% CI: 44.6%‐60.2%), respectively (Figure 1G, *P *= .0407). Fifteen of 87 patients carried the FOLR3 SNP, only two relapsed but they successfully achieved secondary withdrawal by resuming TKI treatment (Table S6).

**FIGURE 1:**
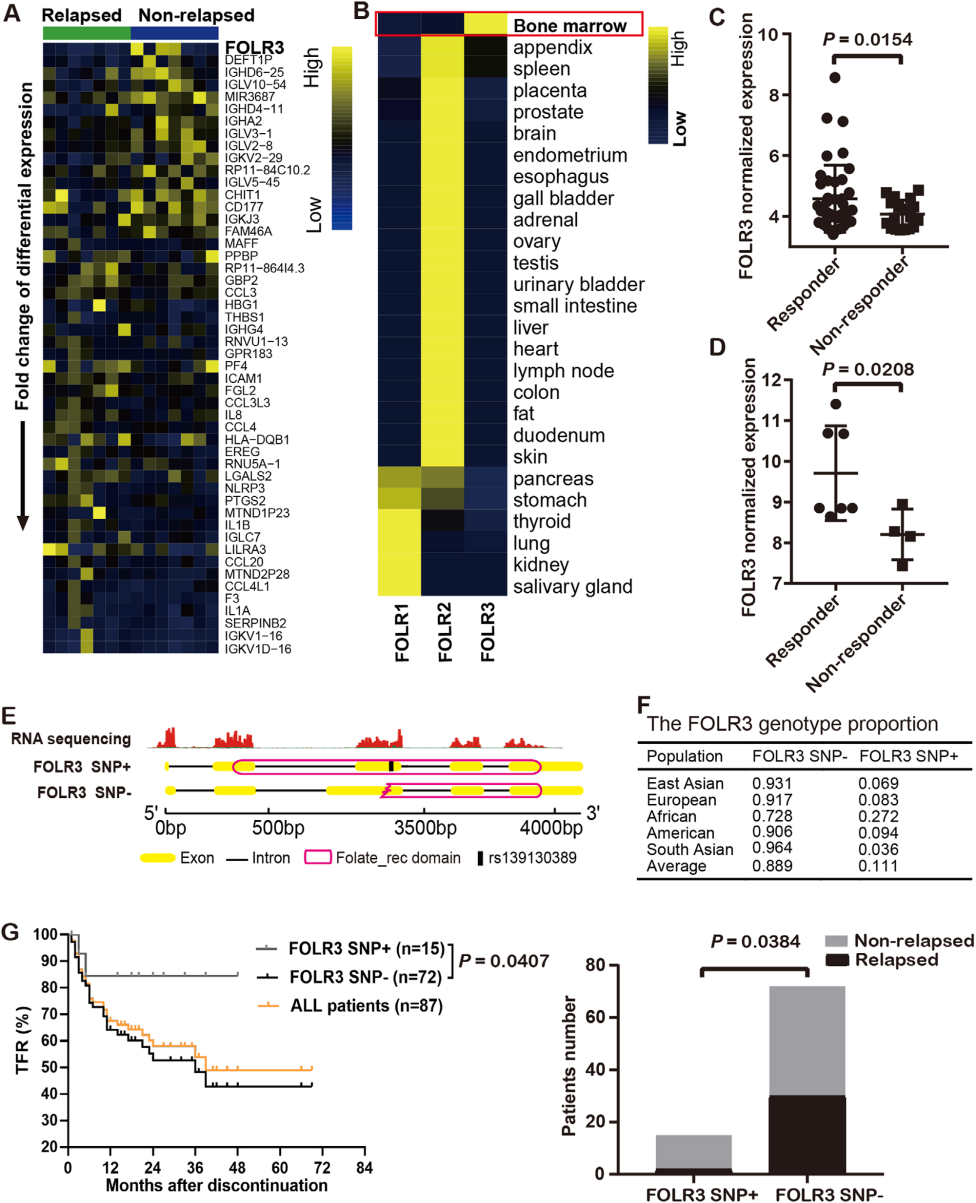
Identifying the differential expression of FOLR3 by an SNP between relapsed and non‐relapsed CML patients. A, Heatmap of the top 50 DEGs from differential expression analysis between relapsed and non‐relapsed CML patients. B, Expression profiles of folate receptor genes in normal tissues from HPA data. C, D, Normalized expression of FOLR3 in responder and non‐responder CML samples after imatinib therapy in datasets GSE14671 and GSE2535. E, The FOLR3 mapping results and their encoded proteins. The TA deletion (SNP+) transcript of FOLR3 encodes a protein with a complete folate receptor domain. F, The SNP rs139130389 genotype frequencies in different populations from the 1000 Human Genome Project. G, TFR and distribution of relapse and non‐relapsed of FOLR3 SNP+/‐ CML patients after TKI discontinuation [n = 87, Log‐rank (Mantel‐Cox) test and likelihood ratio test].

The distributions of immune cells were not significantly different between relapsed and non‐relapsed samples in our cohort (Figure S1).^7^ Therefore, we established different FOLR3 SNP expression subtypes in CD34^+^ cells from newly diagnosed CML patients and K562 cells through lentiviral transfection (details in Supporting Information). The proliferation, cell cycle (Figure 2A; Figure S2), colony‐forming capacities (Figure 2B) and capacity of folic acid uptake (Figure 2C) of FOLR3 SNP+ CD34+ and K562 cells were higher than those of control, siFOLR3 and FOLR3 SNP‐ groups. Folate‐free medium could cancel the difference of colony‐forming units among different groups of K562 cells (Figure 2D). Besides, we found FOLR3 SNP+ K562 cells exhibited the lowest *BCR‐ABL1* expression in the four groups (Figure 2E) but higher sensitivity to TKI (Figure 2H). Metabolomics analysis of FOLR3 SNP+/‐ CD34^+^ indicated they were enriched in fatty acid metabolism, biosynthesis, and elongation pathways (Figure 2F). The glycerophospholipid and fatty acid biosynthesis were more active in FOLR3 SNP+ K562 cells (Figure 2F). Subcutaneous tumorigenesis by K562 cells and small animal PET scanning were performed on three representative mice of each group at 21 days after engraftment. An increase in the maximal standard uptake value of ^18^F‐FDG was found in FOLR3 SNP+ group (Figure 2G).

**FIGURE 2:**
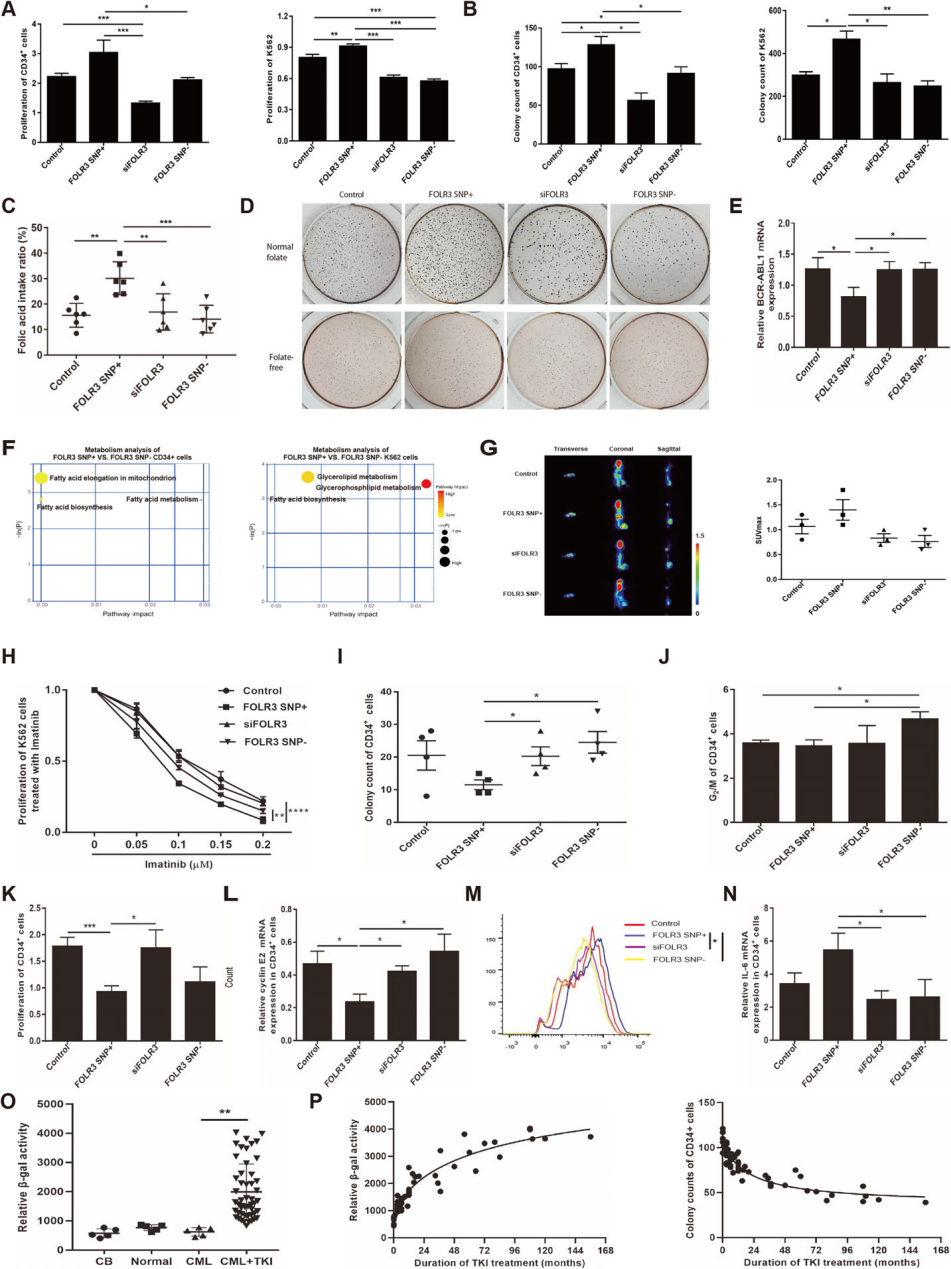
FOLR3 SNP affected the proliferation, metabolism and TKI sensitivity of CML cells. A‐B, Cell proliferation (A) and (B) of CML CD34^+^ and K562 cells with different FOLR3 SNP. C, Capacity of folic acid intake in K562 cells with different FOLR3 SNP. D, Colony images of K562 cells with different FOLR3 SNP cultured in normal folate or folate‐free medium. E, The relative BCR‐ABL1 mRNA expression in K562 cells with different FOLR3 SNP were determined by qRT‐PCR. The results are presented as 2^−ΔΔct^. F, Pathway analysis of differential metabolite upregulated in FOLR3 SNP+ CD34^+^/K562 cells and simultaneously downregulated in FOLR3 SNP‐ CD34^+^/K562 cells. The *x* and *y* axes represent the pathway impact and enrichment, respectively. Larger size and darker color represent increased pathway enrichment and higher pathway impact values, respectively. G, Representative 18F‐FDG PET images and quantification of tumor 18F‐FDG uptake in mice 3 weeks after subcutaneous injection of K562 cells with different FOLR3 SNP (n = 3 images per group). SUV, standardized uptake value. H, Cell proliferation was assessed using the CCK‐8 assay on K562 cells with different FOLR3 SNP treated with imatinib for 48 h. The following experiments were performed 21 days after transfection: I, The statistics for colony numbers for quartic experiments on CD34+ cells with different FOLR3 SNP. J, G2/M stage of CD34^+^ cells were stained by PI and calculated on flow cytometry. K, The proliferation of CD34+ with different FOLR3 SNP was determined by CCK‐8 after 48 h. L, qRT‐PCR was applied to quantify the relative expression of senescence‐associated genes Cyclin E2 in CD34^+^ cells. M, Representative histograms of DCFH‐DA labelled CD34^+^ cells with different FOLR3 SNP. ROS in CD34^+^ cells was determined from the median fluorescence intensity of DCFH‐DA labelled cells. N, qRT‐PCR was applied to quantify the relative expression of senescence‐associated genes IL‐6 in CD34^+^ cells. O, The mean fluorescence intensity (MFI) of SPiDER‐β Gal labelled CD34+ cells from human cord blood (CB), healthy mobilized peripheral blood (Normal), non‐treated CML cells (CML) and CML patients treated with TKI (CML+TKI) were measured on flow cytometry. P, The association analyses between duration of TKI treatment and senescence/colony forming ability of CML CD34^+^ cells. ^*^ *P* < .05, ^**^ *P* < .01, ^***^ *P* < .001

To explore the outcome of continuously proliferated cells, we conducted colony forming assay on cells cultured for 21 days after lentivirus transfection. The colony‐forming capacity of FOLR3 SNP+ CD34^+^ cells remarkably decreased compared to that of siFOLR3 and FOLR3 SNP‐ cells (Figure 2I). The percentages of FOLR3 SNP‐ CD34^+^ cells at G2/M stage were higher than that of the FOLR3 SNP+ counterpart (Figure 2J). Compared with the other arms, the proliferation (Figure 2K), CyclinE2 and p21 (Figure 2L, FigS3) of FOLR3 SNP+ CD34^+^ cells noticeably decreased, while ROS levels (Figure 2M) and senescence‐associated secretory phenotype‐related genes IL‐6 and MMP9 substantially increased (Figure 2N, Figure S3). Compared with CD34^+^ cells from cord blood, healthy mobilization and non‐treated CML patients, we found that CD34^+^ cells in CML patients treated with TKI presented more senescence phenotypes (*P *< .05) (Figure 2O). The longer TKI treatment lasted, the higher of β‐gal activity exhibited, and the fewer colonies produced (Figure 2P).

To explore the mechanism, we performed RNA‐seq for CD34+ cells from 3 newly diagnosed CML patients and K562 cells (Figure 3A), which were transfected with lentivirus to differentially express FOLR3 SNP (blank, FOLR3 SNP+, FOLR3 SNP‐ and siFOLR3, Table S3). The 220 upregulated DEGs in FOLR3 SNP+ CD34+ vs blank comparison were significantly enriched in mitochondrion‐related processes, such as ATP hydrolysis, ATPase activity and mitochondrial electron transport (Figure 3B; Figure S4C). Similarly, 229 upregulated DEGs identified in the FOLR3 SNP+ K562 versus blank comparison were also enriched in mitochondrion‐related processes (Figure 3B). Besides, the FOLR3 SNP+ groups had significantly higher mitochondrion‐related gene expression, as well as oxidative phosphorylation, ATP synthesis and ROS production (Figure 3C).

**FIGURE 3:**
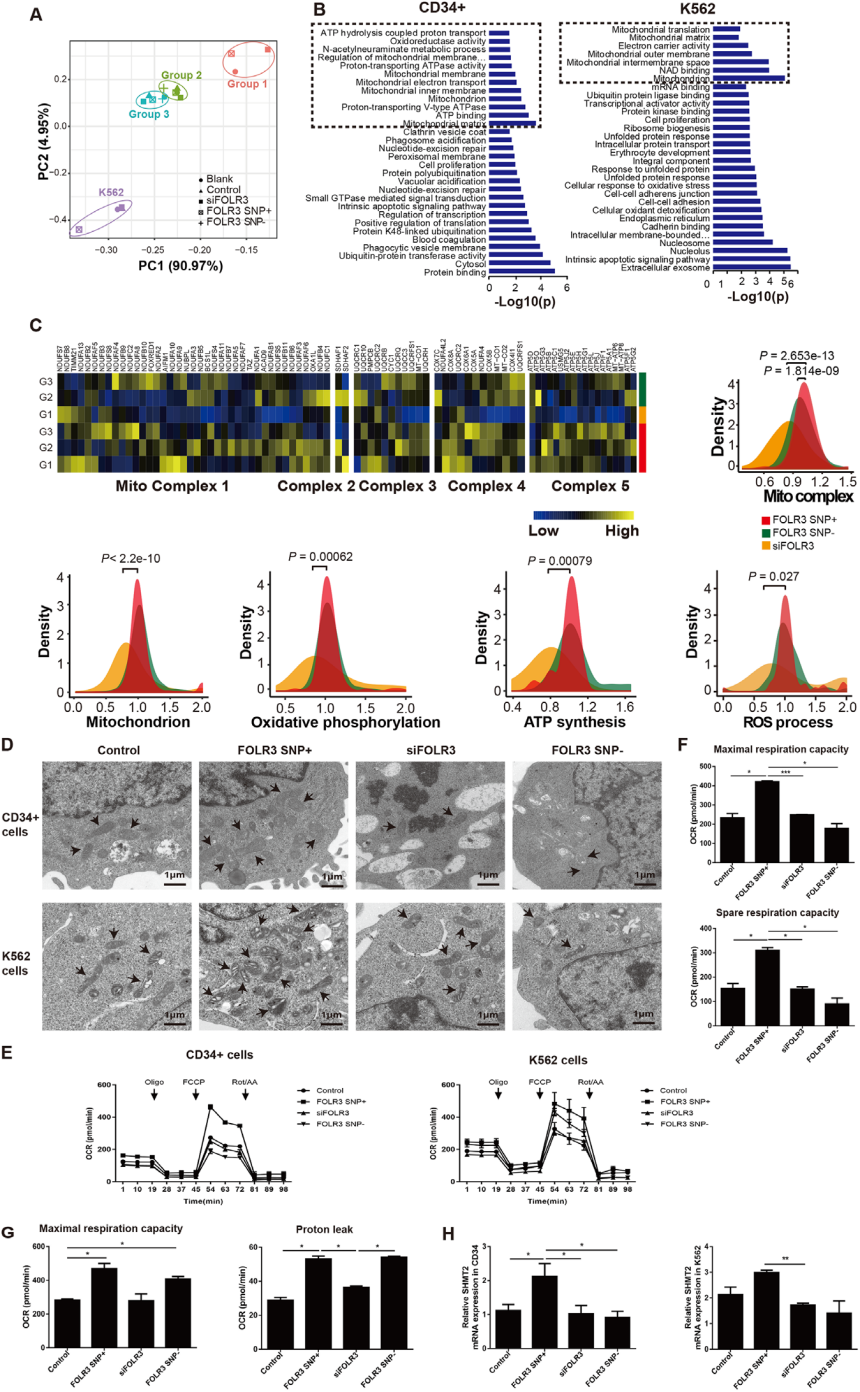
Identifying mechanism of the FOLR3 SNP by promoting mitochondrial function. (A) PCA analysis of gene expression in the five groups (Blank, Control, siFOLR3, FOLR3 SNP^+^ and FOLR3 SNP‐). B Mitochondrion‐related processes were enriched in upregulated DEGs from comparisons of FOLR3 SNP+ vs. blank in all three CD34+ samples and K562 cell lines. (C) Relative expression profiles of mitochondrial complexes and gene expression density of mitochondrial complexes, all mitochondrial genes, oxidative phosphorylation, ATP synthesis and ROS process in FOLR3 SNP+, FOLR3 SNP‐ and siFOLR3 groups. Due to individual differences, gene expression activity (x‐axis) was calculated as the FPKM of experimental group samples (FOLR3 SNP+, FOLR3 SNP‐ and siFOLR3) divided by the FPKM of the blank sample for each group. The *y*‐axis is the density of gene numbers, and its distribution was scaled to the same maximum height. D, Representative electron microscopy images of mitochondria in CD34^+^ cells and K562 cells with different FOLR3 SNP. Magnification 5000× , scale bar = 1 μm. E, Cellular OCRs in CD34^+^ and K562 cells with different FOLR3 SNP. Arrows, the time when oligo (oligomycin), FCCP [carbonyl cyanide‐4‐(trifluoromethoxy) phenylhydrazone], and Rot/AA (antimycin/rotenone) were added to the system. Data were obtained using the Seahorse XF24 analyzer. F, Quantified indexes, maximal respiration capacity and spare respiration capacity of CD34^+^ cells with different FOLR3 SNP were calculated by the Seahorse XF24 analyzer. G, Maximal respiration capacity and proton leakage in K562 cells with different FOLR3 SNP were quantified by the Seahorse XF24 analyzer. H, SHMT2 mRNA levels in CD34^+^ and K562 cells with different FOLR3 SNP were determined by qRT‐PCR. The results are presented as 2^−ΔΔct^. *P* values were tested by the paired t‐test. ^*^ *P* < .05, ^**^ *P* < .01, ^***^ *P* < .001

Transmission electron microscopy indicated that FOLR3 SNP+ K562 cells exhibited rich amounts of lipid droplets, which were not observed in SNP+ CD34+ cells (Figure 3D). The FOLR3 SNP‐ groups had fewer mitochondria than SNP+ groups (Figure 3D). FOLR3 SNP+ CD34^+^ and K562 cells had higher oxygen consumption rates (Figure 3E), maximal respiration and spare respiratory capacity (Figure 3F; Figure S5A). In K562 cells, FOLR3 SNP significantly increased maximal respiration and proton leakage; however, differences in basal respiration between each group were not notable (Figure 3G; Figure S5A). Notably, mitochondrial membrane potential and ATP concentrations of FOLR3 SNP+ CD34^+^ cell was significantly higher (Figure S5B, C). Serine hydroxymethyltransferase 2, a key enzyme in folate‐dependent mitochondrial translation and oxidative phosphorylation,^8^ highly expressed in SNP+ CD34^+^ and K562 cells (Figure 3H; Figure S5D).

In conclusion, we detected FOLR3 SNP rs139130389 only in the TFR group. FOLR3 SNP+ CML cells proliferated actively and exhibited greater colony‐forming ability via elevating mitochondrion activity. Proliferating cells were relatively lower BCR‐ABL1 but more sensitive to TKI. Further, continuous proliferation of stem cells would induce replicative senescence.^9^ We speculate patients achieve TFR because their aging CML‐LSCs failed to produce malignant clones after discontinuation. As a result, CML‐LSCs senescence might be a key point of discontinuation and the time needed to take medicine would be personalized for CML‐LSCs to accumulate senescence. The idea of senescence will provide an outlook on future challenges of CML‐LSCs elimination.

## CONFLICT OF INTEREST

The authors declare no conflict of interest.

## FUNDING

This work was supported by the National Natural Science Foundation of China (31822030 and 31771458 to A.Y.G., 81500136 to X.Z., 81700142 to Q.L., 81873440 to Y.Y.), Key R & D plan of Hubei Province (No.2020BCB021 & 2020BCB043).

## AUTHOR CONTRIBUTIONS

X.J.Z., A.Y.G., and Y.Y. designed the research and reviewed the paper; N.S., X.Y.Z., W.L., and T.L. performed experiments; T.L., A.Y.G., Q.L., and N.S. analyzed results and made the Figures; X.J.Z., A.Y.G., and N.S. wrote the paper; W.L. and Z.D.Z. interpreted data and reviewed the paper. P.Z. provide the materials and reviewed the paper.

## Supporting information


**SUPPORTING INFORMATION**: Additional supporting information may be found online in the Supporting Information section at the end of the article.
