# Supplementary material for: A folate receptor 3 SNP promotes mitochondria‐induced clonogenicity of CML leukemia cells: Implications for treatment free remission
Source: Clin Transl Med. 2021 Feb 4;11(2):e317. doi: 10.1002/ctm2.317 (PMC7862584; doi:10.1002/ctm2.317)
Supplement: Supplementary file 1 — SUPPORTING INFORMATION: Additional supporting information may be found online in the Supporting Information section at the end of the article. [file CTM2-11-e317-s001.docx]

**SUPPLEMENTARY INFORMATION**

**A Folate Receptor 3 SNP Promotes Mitochondria-Induced Clonogenicity of CML Leukemia Cells: Implications for Treatment Free Remission**

The ethical of this work was reviewed by the ethics committee of Tongji Medical College, Huazhong University of Science and Technology. More detailed descriptions of the materials and methods were shown in Supplemental Materials.

1. **Materials and Methods**

**1.1 Patient selection and criteria**

The inclusion criteria of discontinuation were as follows: (1) a confirmed diagnosis of CML in the chronic phase; (2) ongoing treatment with imatinib at a standard dose for at least 3 years or switching to a second-generation TKI for at least 1 year; (3) sustained complete molecular response for at least 1 year before TKI cessation; (4) no history of allogeneic hematopoietic stem-cell transplantation, and (5) attendance of follow-up molecular biological tests. Molecular relapse was defined as loss of major molecular response (MMR).

BM samples for sequence were extracted from 14 CML patients just before discontinuation following the principle of informed consent. The average age of these patients was 35.67 year (range, 19-58 year). The average time to achieve a MMR was 9.13 months (range, 3-14 months). The average duration of TKI treatment was 85.41 months (range, 77-103 months). A 24 months’ period of relapse/TFR was set as the endpoint of this observation, and samples from these patients were divided into relapse and TFR groups.

We also retrospectively studied 87 CML patients, who discontinued TKI treatment outside of clinical trials due to any reason between June 2012 and December 2019 in 8 authoritative hospitals in China. The eligibility criteria were consistent with those described above. All samples underwent Sanger sequencing to detect FOLR3 SNP. In the case of molecular relapse, patients were recommended to immediately resume TKI treatment.

Samples from 112 CML patients for Sanger sequencing were taken from randomized CML-CP patients, regardless of gender, age, Sokal score, newly diagnosed or under treatment and remission status etc.

Normal mobilized peripheral blood samples were obtained from Institute of Hematology, Union Hospital (Wuhan, China). Human cord blood samples were obtained from full-term pregnant women who underwent caesarean section at Department of Gynecology and Obstetrics, Union Hospital (Wuhan, China). All samples were collected with the consent of the patients and the approved by the ethics committee of Tongji Medical College, Huazhong University of Science and Technology.

**1.2 Bioinformatics analyses for RNA-seq data, expression, and function**

HiSeq2500 (Illumina, Inc. San Diego, USA) mRNA sequencing was performed at BGI (Shenzhen, China). After filtering, clean reads were mapped to the human hg19 reference genome using STAR alignment. ^15^ The levels of gene expression were normalized by FPKM with StringTie. ^16^ The NOISeq package was used to identify the differentially expressed genes (DEGs) between 7 CML cases with and 7 cases without molecular relapse (Table S2). DEGs among cells of FOLR3 SNP+ (with the SNP rs139130389), FOLR3 SNP- (wild-type FOLR3, without the SNP) and siFOLR3 (cells transfected with an siRNA of FOLR3) groups were separately examined in the CML CD34+ and K562 groups (Table S3). The GATK package ^17^ was applied to call SNPs from the mRNA sequencing data with default parameters. Enrichment analyses of all DEGs were performed by using the DAVID database. ^18^ Principal component analysis (PCA) was conducted by using the R and ggplot2 packages. The mRNA and protein sequences for FOLR3 with or without the TA insertion at rs139130389 were obtained from UCSC Genome Browser. ^19^ The GSE14671 ^20^ and GSE2535 ^21^ datasets were downloaded from the GEO database. MCP-counter ^22^ and ImmuCellAI ^23^ were used to count stromal and immune cell populations for our expression data. In order to perform pathway expression activity analysis, we collected the mitochondrion-related genes according to the methods that were described in Table. S4. We also applied the relative expression value for each gene and draw a heatmap and density profile of the mitochondrion-related processes for the CD34+ group with the ggplot2 package.

**1.3 Cell culture**

CD34+ cells used for the functional studies were from newly diagnosed chronic phase CML patients following the principle of informed consent. The CD34+ stem and progenitor cells were isolated using a CD34 MicroBead Kit (Miltenyi Biotech, Cologne, Germany) and cultured in serum free medium StemSpan (StemCell Technologies, Vancouver, Canada) with SCF (100 ng/ml, PeproTech, Rocky Hill, USA), FLT-3-L (100 ng/ml, PeproTech, Rocky Hill, USA), IL-3 (20 ng/ml, PeproTech, Rocky Hill, USA), and IL-6 (20 ng/ml, PeproTech, Rocky Hill, USA). A human CML cell line, K562, was purchased from and authenticated by China Center for Type Culture Collection (Wuhan, China). The authentication result matched the STR data of cell lines in the databases of ATCC repositories DSMZ and JCRB.

**1.4 Cell transfection**

CD34+ and K562 cells (2 × 10^5^) were pre-seeded onto plates coated with RetroNectin (Takara, Japan) overnight. Lentiviral vectors (Genechem, Shanghai, China) were added to the cell suspension (multiplicity of infection of CD34+ and K562 cells were 150 and 50, respectively) and incubated for 16h for CD34+ cells or 10h for K562 cells before changing the medium. Two days later, transfection efficiency was identified by fluorescence microscope. The transfection efficiency of CD34+ and K562 cells was 20% and 80%, respectively (Supplementary Figure 1A), and PCR was performed to verify the relative expression of endogenous and exogenous FOLR3 (Supplementary Figure 1B). Then, purinomycin was added to the medium to screen the successfully transfected cells. Each experiment was repeated at least three times.

**1.5 Colony-forming cell assay**

**For CD34+ cells, 1500 cells were suspended in 1 ml MethoCult^TM^ H4435 Enriched (StemCell Technologies,** Vancouver**, Canada) and then seeded in 24-well plates in duplicate. Surrounding wells were supplemented with 1 ml PBS. Clones were scored after 10-14 days of incubation at 37°C and 5% CO2. For K562 cells, 3 000 cells were suspended in 1 ml 0.33% soft agar (****BBI Life Sciences, Shanghai, China) mixed with cell culture medium that rested on another layer of 2 ml 0.5% soft agar also mixed with cell culture medium in a 6-well plate. Clones were stained with MTT and scored after 10-14 days of incubation at 37°C and 5% CO2.**

**1.6 qRT-PCR**

Total RNA was extracted using Trizol reagent (Takara, Dalian, China). cDNA was synthesized by reverse transcription of 500 ng total RNA using a PrimeScript^TM^ RT Reagent Kit (Takara, Dalian, China) following the procedure: 37°C for 15 min, 85°C for 5 s and hold in 4°C. Real-time PCR was performed with 5 μl SYBR Premix Ex Taq (Takara, Dalian, China), 0.8 μl primers, 0.2 μl ROX Reference Dye (Takara, Dalian, China), 3 μl RNase-free H_2_O and 1 μl cDNA as a template in a final reaction volume of 10 μl. The control gene was GAPDH. The PCR cycling conditions were as follows: initial melting at 95°C for 30 sec, followed by 40 cycles at 95°C for 5 sec and 60°C for 30 sec. Melting curve was performed at 95°C for 15 sec, 60°C for 60 sec and 95°C for 15 sec to determine the specificity of primers. Fluorescence intensity was measured using the ABI StepOnePlus^TM^ Real Time PCR System (Waltham, MA, USA). Analysis of the melting curve for the primers was conducted to confirm the specificity of the PCR product, and the Ct value for triplicate reactions was averaged. The fold changes in mRNA were calculated through relative quantification (2^-ΔΔCt^).

**1.7 Apoptosis detection**

K562 and CD34+ cells (both 1 × 10^5^) were harvested by centrifugation and washed twice with ice-cold PBS. Then, the cells were stained with Annexin-V-FITC/PI (BD Pharmingen) for 15 min at room temperature. All analyses were carried out on a flow cytometer (BD LSRFortessa^TM^ X-20). The results were analyzed on FlowJo V10 (Stanford University, San Francisco, CA, USA).

**1.8 Cell cycle analysis**

CD34^+^ and K562 cells (both 3 × 10^5^) were fixed with 70% absolute ethyl alcohol overnight. Then, the cells were washed and stained with a mixture of PI and RNAase (BD Pharmingen, Franklin Lake, NJ, USA) at 37°C for 30 min and analyzed via flow cytometry (BD LSRFortessa^TM^ X-20). The results were analyzed using ModFit LT software (Verity Software House, ME, USA).

**1.9 Cell proliferation assay**

CD34+ and K562 cell proliferation were measured through CCK-8 (Biosharp, Hefei, China). A total of 1 × 10^4^ cells were plated in quintuplicate in 96-well plates and incubated for 48 h. CCK-8 reagent (10 ul) was added to the plates, followed by incubation for 2-4 h at 37°C and 5% CO_2_. The absorbance (450 nm) was measured on a multifunctional enzyme marking instrument (Perkin Elmer Singapore Pte. Ltd Singapore).

**1.10 Folic acid intake assay**

K562 cells were cultured in folic acid-free RPMI 1640 (Gibco, California, USA) supplemented with FITC-folic acid (H&P, China) according to the manufacturer’s instructions for 48h in dark. Then, cells were harvested and washed twice with PBS in dark. Finally, cells were detected using flow cytometry (BD LSRFortessa^TM^ X-20). The results were analyzed on FlowJo V10 (Stanford University, San Francisco, CA, USA).

**1.11 Positron emission tomography (PET) imaging**

The K562 cells that differentially express the FOLR3 SNP were collected and subcutaneously injected into 4 to 5-weeks-old immuno-deficient BALB/c nude mice (Beijing Vital River Laboratory Animal Technology Co., Ltd., Beijing, China). On the 21st day after subcutaneous engraftment, the mice were fasted and anesthetized with 2% isoflurane. Approximately 200 ± 20 μCi of 18-fluoro-6-deoxy-glucose (18F-FDG) was injected via the tail vein. Static PET images were started 1 h after the injection using a Trans-PET BioCaliburn LH system (Raycan Technology Co., Ltd. Suzhou, China). A volume-of-interest analysis was conducted using the AMIDE software package (The Free Software Foundation, Inc., Boston, USA).

**1.12 Analysis of SA-β gal activity via flow cytometry**

SA-β-gal activity was detected using a Cellular Senescence Detection Kit-SPiDER-β Gal (Dojindo, Shanghai, China). CD34+ cells (1×10^5^) were treated with Bafilomycin A1 for 2 hours at 37℃, 5% CO_2_, then the cells were stained with SPiDER-β Gal for 30 min at 37℃, 5% CO_2_. All analyses were carried out on a flow cytometry (BD LSRFortessa^TM^ X-20). The results were analyzed on FlowJo V10 (Stanford University, San Francisco, CA, USA).

**1.13 Ultra-performance liquid chromatography mass spectrometry on metabonomics**

CD34+ and K562 cell pellets (~2 × 10^6^) were collected in an EP tube, and 1000 μl extraction liquid (acetonitrile-methanol-water, 2:2:1, containing internal standard) were added, the samples were vortexed for 30 sec, homogenized at 45 Hz for 4 min, and sonicated for 5 min in an ice-water bath. The homogenization and sonication cycle was repeated 3 times, followed by incubation at -20°C for 1 h and centrifugation at 12,000 rpm and 4°C for 15 min. The resulting supernatants were transferred to LC-MS vials and stored at -80°C until LC-MS analyses using an UHPLC system (1290, Agilent Technologies, Santa Clara, CA, USA). MS raw data files were converted to the mzML format using ProteoWizard (Palo Alto, CA, USA) and processed by R package XCMS (version 3.2, La Jolla, CA, USA). The preprocessing results generated a data matrix that consisted of the retention time, massto-charge ratio (m/z) values, and peak intensity. OSI-SMMS (version 1.0, Dalian ChemDataSolution Information Technology Co., Ltd.) was used for peak annotation after XCMS data processing with an in-house MS/MS database. PCA and orthogonal projections to latent structures-discriminant analysis (OPLS-DA) were done using SIMCA-P 14.1 software package (Sartorius Stedim Data Analytics AB, Umea, Sweden) to obtain reliable metabolite differences between FOLR3 SNP+ and FOLR3 SNP- CD34+/K562 cells. The standards for differentially expressed metabolites screening were variable importance on the projection (VIP, obtained from OPLS-DA model) > 1 and p (obtained from student’s t-test between FOLR3 SNP+ and FOLR3 SNP- CD34+/K562 cells) < 0.05. The Kyoto Encyclopaedia of Genes and Genomes (http://www.genome.jp/kegg/) and MetaboAnalyst 3.0 (http://www.metaboanalyst.ca/) were utilized to analyze the metabolic pathways.

**1.14 Transmission electron microscopic scanning**

K562 and CD34+ cells (~2 × 10^6^) with different the FOLR3 SNP were collected in EP tubes and fixed with 2.5% glutaral overnight. Transmission electron microscopic scanning was performed by the Core Facility and Technical Support, Wuhan Institute of Virology.

**1.15 Cell mitochondrial stress test**

The cell mitochondrial stress tests were executed using Seahorse XF Cell Mito Stress Test Kit (Agilent Technologies, Santa Clara, USA) and analysed on XF24 extracellular flux analyzer (Seahorse Bioscience, MD, USA) according to the methods that are described previously. ^24^ Briefly, the XF24 cell culture microplates were coated with Cell-Tak (Corning, New York, USA), and were seeded at 2 × 10^5^ per well for CD34+ cells and at 7 × 10^4^ per well for K562 cells. Data were analyzed using Wave software 2.6 (Seahorse Bioscience, MD, USA).

**1.16 Mitochondrial membrane potential**

To measure mitochondrial membrane potential, cells were incubated with 500 ul 5,5',6,6'-tetrachloro-1,1',3,3'-tetraethyl benzimidazolylcarbocyanine iodide (JC-1, Beyotime Biotechnology) working solution for 20 min at 37°C. Then, all samples were washed and resuspended in PBS prior to being read by flow cytometry (BD LSRFortessa^TM^ X-20). Data were analyzed with FlowJo version 10 (Stanford University, San Francisco, CA, USA).

**1.17 Drug treatment and ATP detection**

K562 cells were treated with AICAR (Selleck, Shanghai, China) and DAC (Selleck, Shanghai, China) at different concentration gradients for 48 h. Cells (1×10^5^) were collected and intracellular ATP was determined using an ATP assay kit (Beyotime Biotechnology, Shanghai, China) according to the manufacturer’s instructions.

**1.18 Statistical analysis**

Data were analyzed using GraphPad Prism 8.0.1 (GraphPad Prism Inc., La Jolla, USA), and P < 0.05 was accepted as statistically significant. Data are presented as the mean ± SEM. The RNA-seq data are available at BIGD Genome Sequence Archive under accession numbers PRJCA001071 and PRJCA001072.

**2．Supplementary and more detailed Results**

As letter described, we sequenced BM mononuclear cells of 7 relapsed and 7 non-relapsed CML patients. The median cumulative duration of TKI treatment was 79.5 months (range, 54-95 months) and the median time to achieve a major molecular response was 9 months (range, 3-24 months). The median time of relapse was 4 months (range, 1-14 months); among them, 5/7 of patients had relapsed in 6 months after drug discontinuation (Table S1). PCA analysis of the two groups showed large individual differences (Fig S1A). We also analyzed the DEGs between these two groups. In addition to FOLR3, we also noted that the FOLR1 and FOLR2, the other two genes in the FOLR family, were not expressed in any of these samples (Fig S1B). Based on Genotype-Tissue Expression (GTEx, Fig S1C), we found that the FOLR3 was also highly expressed in blood. To evaluate the FOLR3 expression in other CML cohorts, we collected two CML datasets, as follows: GSE14671, comprising 59 CML CD34+ cell samples with a complete cytogenetic response (responders) after 12 months of imatinib therapy and 18 lacking a major cytogenetic response (non-responders)]; GSE2535, with 11 CML BM samples (7 responders after imatinib therapy and 4 non-responders). As described in letter, the FOLR3 was highly expressed in the responders of both datasets (Fig 1C, D). These findings indicate that the FOLR3 may be beneficial to CML therapy. The TCGA cancer data showed that patients in AML or other non-CML tumors with a high level of FOLR3 expression had poor overall survival rate (Fig S1D, E), suggesting that the role of FOLR3 in CML may be specific. The expression level of FOLR3 was positively correlated with the number of mapped reads with the TA insertion at rs139130389 (Fig S1H). In the three FOLR3 SNP+ samples, nearly 100% of the reads mapped on rs139130389 showed the TA insertion, indicating that the SNP rs139130389 is homozygosity. Based on evolutionary analysis, we found that the TA allele of SNP rs139130389 occurred in almost all non-primate species but that it is absent in most primates (Fig S1I). We collected 112 chronic phase CML samples for Sanger sequencing, ten of which were FOLR3 SNP+ genotypes (8.93%, Table S5). As described in letter, we also retrospectively studied 87 CML patients who discontinued TKI treatment outside of clinical trials for any reason. The median follow-up time was 14 months (range, 1-69 months). Among 72 patients without the FOLR3 SNP, 30 patients relapsed and 17/30 (56.67%) relapsed within 6 months after discontinuation. For all of the 87 patients, the ratio of TFR was 67.6% (95% CI: 62.2%-73.0%) at 12 months, 58.0% (95% CI: 51.9%-64.1%) at 24 months, and 49% (95% CI: 41.1%-56.9%) at 48 months (Fig 1G).

As described in letter, we established different FOLR3 SNP expression subtypes in CD34+ cells from newly diagnosed CML patients and K562 cells through lentiviral transfection. In brief, the blank group were cells without any intervention; control cells were transfected with lentivirus containing a scrambled sequence; the FOLR3 SNP+ group consisted of cells transfected with lentivirus carrying the FOLR3 SNP sequence; the siFOLR3 group cells transfected with a sequence that interfered with the expression of FOLR3 SNP, and the FOLR3 SNP- group comprised cells overexpressing FOLR3, without the SNP. The transfection efficiency was confirmed by fluorescence microscope and qRT-PCR (Fig S2A, B). The FOLR3 SNP had no impact on apoptosis (Fig S2C). The single-clone morphology of CD34+ cells was mainly colony-forming unit-erythroid (Fig S2E). To identify whether FOLR3 SNP promotes colony-forming capacity by increasing folate uptake, we seeded K562 cells into normal folate and folate-free soft agar medium. Although the colony size of the normal folate group was generally larger than that of the folate-free group, there was no significant difference in the number of cell colony-forming units among the folate-free groups with different FOLR3 SNP expression (Fig 2D). Besides, we found FOLR3 SNP+ K562 cells exhibited the lowest BCR-ABL1 expression in the four groups (Figure 2E) but higher sensitivity to TKI (Figure 2H). We speculate that the replication and transcription of housekeeping genes are very active in the proliferative cells, which might exceed the transcription of BCR-ABL1. Therefore, when we calculate the ratio of BCR-ABL1 mRNA to housekeeping genes such as actin, ABL1 or GAPDH, the result indicated that BCR-ABL1 was relatively low. Besides, in an additional study, we found that CML cells with FOLR3 SNP secreted more extracellular vesicles secreting BCR-ABL1 mRNA, which might also contribute to the relatively lower BCR-ABL1 in cells. We conducted in vivo experiments of subcutaneous tumorigenesis by cells in 4 groups (control, FOLR3 SNP+, siFOLR3 and FOLR3 SNP- K562 cell groups as described in the above paragraph) individually and 5 mice in each group. The small animal PET scanning was performed to explore tumor metabolism. An increase in the maximal standard uptake value (SUVmax) of 18F-FDG was noted in the FOLR3 SNP+ group, with a 1.33-fold increase compared with that in the control (P = 0.2594) and a 1.65-fold increase for siFOLR3 (P = 0.0663) and a 1.82-fold increase for FOLR3 SNP- (P = 0.0579); however, differences lacked significance (Fig 2G).

In the colony forming assay on cells cultured for 21 days after lentivirus transfection. In CD34+ cells, colony-forming unit-granulocyte macrophage predominated (Fig S3A). While the colony-forming capacity of K562 cells with different FOLR3 SNP exhibited no difference at d21 (Fig S3B).

To further explore the mechanism of FOLR3 SNP promoting cell differentiation and aging, we performed RNA-seq for BM CD34+ cells from 3 newly diagnosed CML patients (named group 1, 2, 3 in Fig 3A) and K562 cells. PCA demonstrated clear distinctions for each group, especially between the K562 group and the CD34+ groups (Fig 3A). Moreover, the average gene expression statistics indicated there were more highly expressed genes in the K562 group than the CD34+ groups (Fig S4A). After SNP calling on RNA-seq data, we found that among all blank samples, only group 1 had the rs139130389 TA insertion and expressed the FOLR3 SNP (Fig S4B). We identified 428 DEGs (220 upregulated and 208 downregulated) in FOLR3 SNP+ CD34+ samples vs. blank samples. 370 DEGs were identified (229 upregulated and 141 downregulated) in the FOLR3 SNP+ K562 sample vs. blank sample. Furthermore, expression of 13 mitochondrial protein-coding genes was significantly higher in the FOLR3 SNP+ K562 group (Fig S4D). We assessed the relative expression activity of mitochondrial-related genes in FOLR3 SNP+ and FOLR3 SNP- CD34+ samples from group 1, 2 and 3. Exceptionally, we used group 1 for above analyses in siFOLR3 CD34+ samples because only group 1 had the TA insertion at rs139130389 and expressed the FOLR3 SNP. The FOLR3 SNP+ groups had significantly higher mitochondrion-related gene expression, as based on the relative expression of mitochondrial complexes (Fig 3C). In addition, expression density analyses for the mitochondrion (from MitoCarta), oxidative phosphorylation (from KEGG), ATP synthesis (from GO) and ROS process (from GO) revealed that the FOLR3 SNP+ and FOLR3 SNP- CD34+ samples had higher gene expression than did the siFOLR3 samples (Fig 3C).

We also explored whether drug treatment elevates mitochondrial activity and further allows FOLR3 SNP- cells to achieve biological characteristics similar to FOLR3 SNP+ cells. Our pre-experiments showed that 5-aminoimidazole-4-carboxamide ribonucleoside (AICAR) and 5-aza-2'-deoxycytidine (DAC) were able to elevate ATP production in K562 cells in a concentration-dependent manner within certain limits (Fig S5E).

**
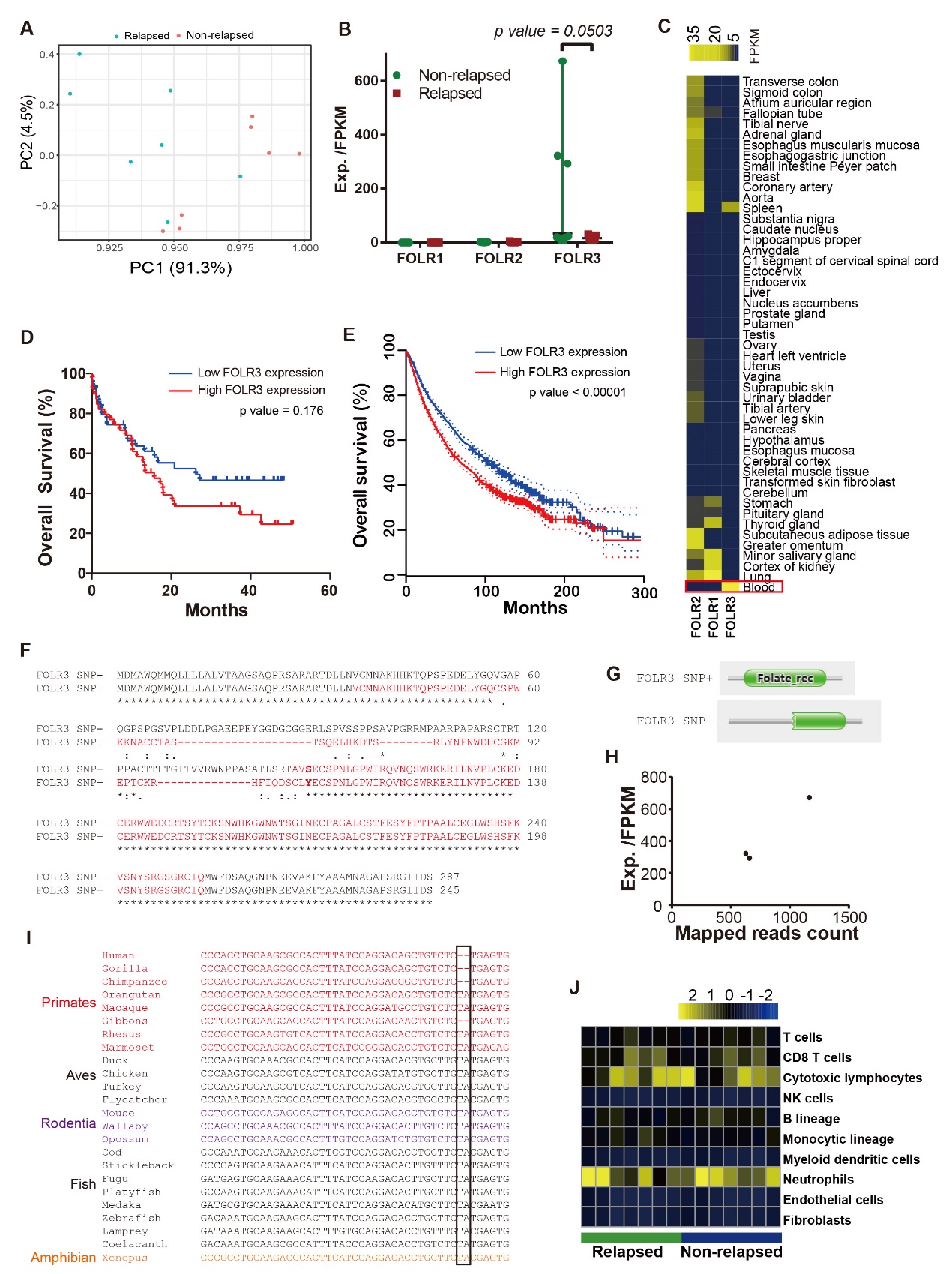
**

**Supplementary Fig. S1.** Identifying FOLR3 SNP and expression level between relapsed and nonrelapsed CML patients. **(A)** The PCA analysis of relapsed and nonrelapsed groups showed that individual differences were large. **(B)** The expressions of FOLR1, FOLR2 and FOLR3 in relapsed and nonrelapsed groups. **(C)** The folate receptor family gene profile from Gtex showed that FOLR3 was especially highly expressed in blood. **(D)** Survival analysis on 78 AML samples from the TCGA LAML dataset. **(E)** Survival analysis on 9346 samples from 33 types of TCGA tumor. **(F)** The protein sequences comparing results, and the red letter was folate receptor domain, the enlarged letter S and Y was the TA insertion and deletion position, we can see that FOLR3 TA Insertion protein sequences has a longer folate receptor domain, and the FOLR3 TA deletion folate receptor domain was started at rs139130389. **(G)** The Pfam domain prediction of FOLR3 TA insertion and deletion sequences. **(H)** The mapped reads count of rs139130389 and the gene expression FPKM of FOLR3 was positive correlation among the three FOLR3 highly expressed samples in nonrelapsed group. **(I)** The FOLR3 gene type of rs139130389 of primates, aves, rodentia, fish and amphibian showed that only primates existed TA deletion in rs139130389. **(J)** The comparison on stromal and immune cell populations in relapsed and non-relapsed patients.

_
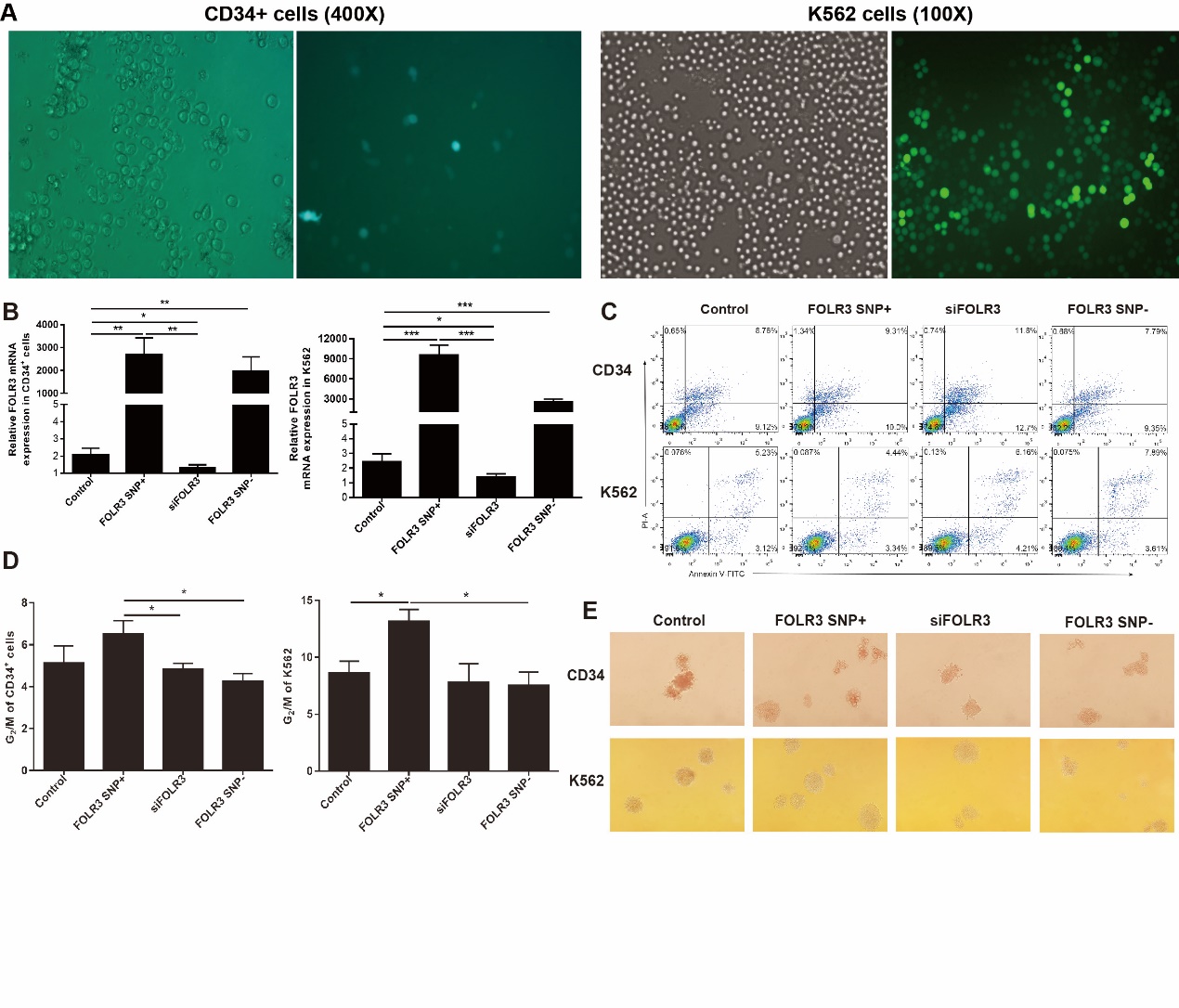
_

**Supplementary Fig. S2.** FOLR3 SNP primarily affected the colony-forming capacity of CD34+ and K562 cells. **(A)** Transfection efficiencies of CD34+ and K562 cells. **(B)** Relative FOLR3 mRNA level were determined by qRT-PCR 3 days after lentivirus transfection in CD34+/K562 cells with different FOLR3 SNP, that is control, FOLR3 SNP+, siFOLR3, FOLR3 SNP- CD34+/K562 cells. **(C)** Three days after transfection, the apoptosis of CD34+/K562 cells with different FOLR3 SNP was investigated with Annexin V/PI staining and quantified by flow cytometry assay. **(D)** Cell cycle analyses of CML CD34+ and K562 cells with different FOLR3 SNP. The meanings for Y-axis is fold change. **(E)** Representative colony images on three days after transfection of CD34+ and K562 cells with different FOLR3 SNP. The images represent one microscope field in each group. Error bars represent the mean ± SEM for three independent measurements. ^*^*P* < 0.05, ^**^*P* < 0.01, ^***^*P* < 0.001.


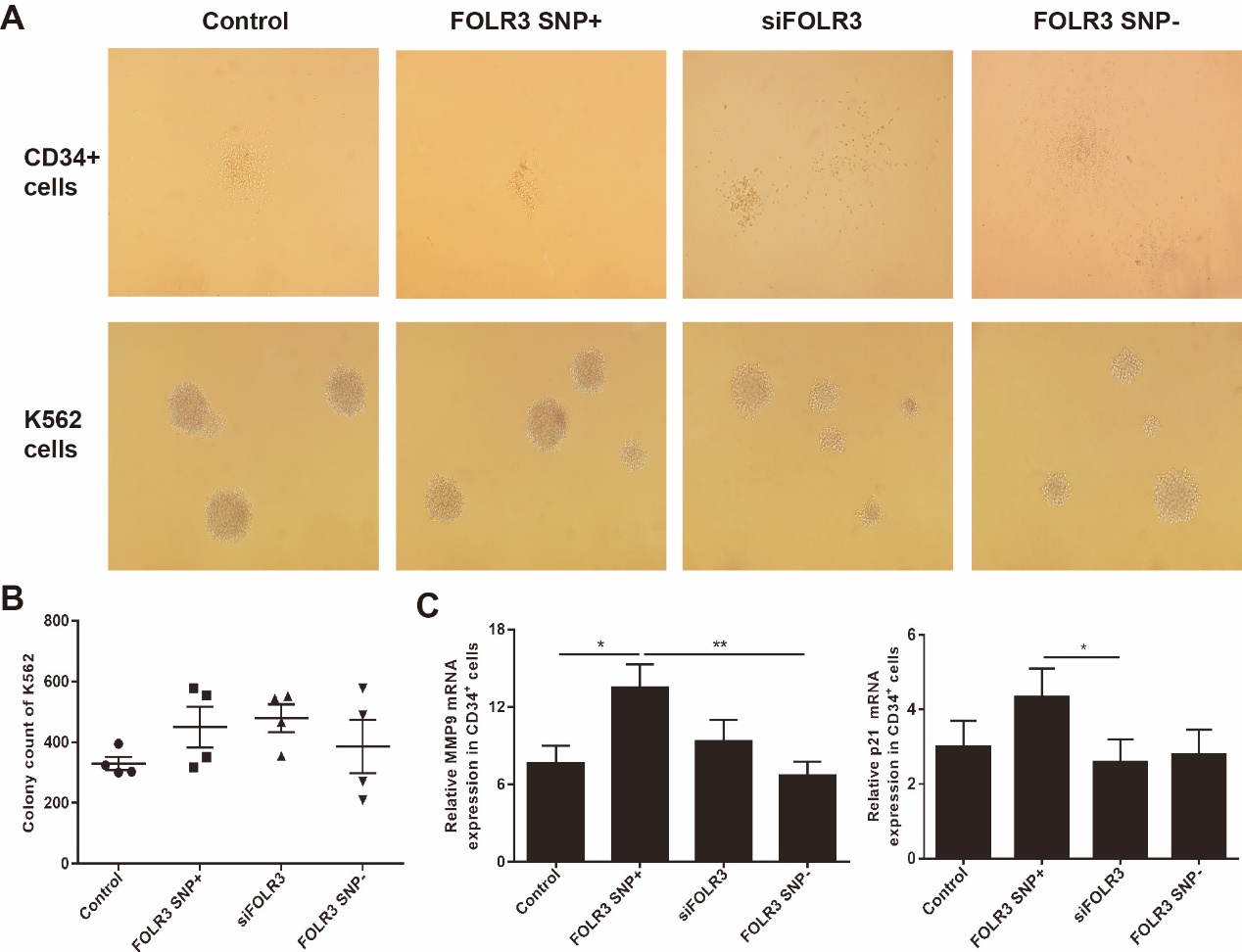


**Supplementary Fig. S3.** CML leukemia cells with continuous differentiation showed senescence phenotype. **(A)** Representative colony images on 21 days after transfection of CD34+ and K562 cells with different FOLR3 SNP. The images represent one microscope field in each group. **(B)** Colony forming assay of K562 cells with different FOLR3 SNP. (C) qRT-PCR was applied to quantify the relative expression of senescence-associated genes MMP9 and p21 in CD34+ cells with different FOLR3 SNP. Error bars represent the mean ± SEM for three independent measurements. ^*^*P* < 0.05, ^**^*P* < 0.01, ^***^*P* < 0.001.

**
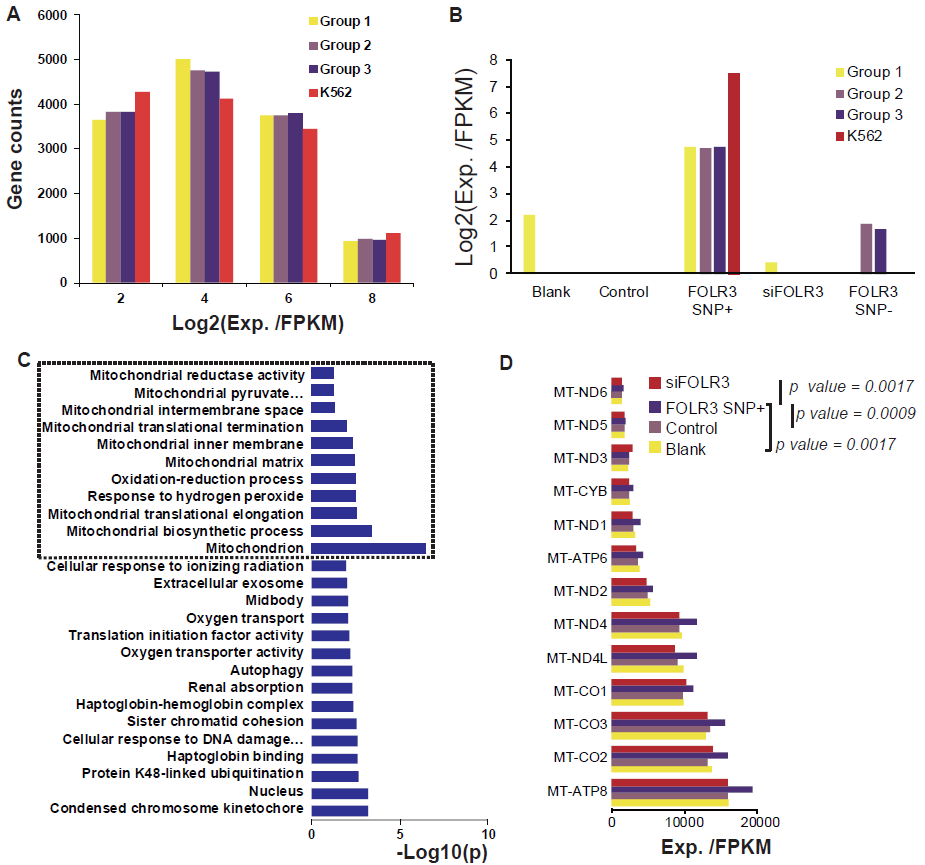
**

**Supplementary Fig. S4.** Mitochondria related pathways and genes were activated in FOLR3 SNP+ cells. **(A)** The gene counts of average expression range showed that K562 group had a different range compared to CD34+ groups. **(B)** The FOLR3 expression of the four groups showed that only group 1 had FOLR3 expressed in blank sample. Due to only Group 1 had TA insertion in rs139130389 and FOLR3 expressed in group 1 blank sample, the next analyses related FOLR3 siRNA was based on the group 1 siRNA. **(C)** The mitochondrial terms were enriched in GO enrichment of 99 upregulaed DEGs CD34+ group FOLR3 TA deletion transcript mimics. **(D)** The expression of 13 mitochondrial protein-coding genes was significantly higher in the FOLR3 SNP+ K562 group.


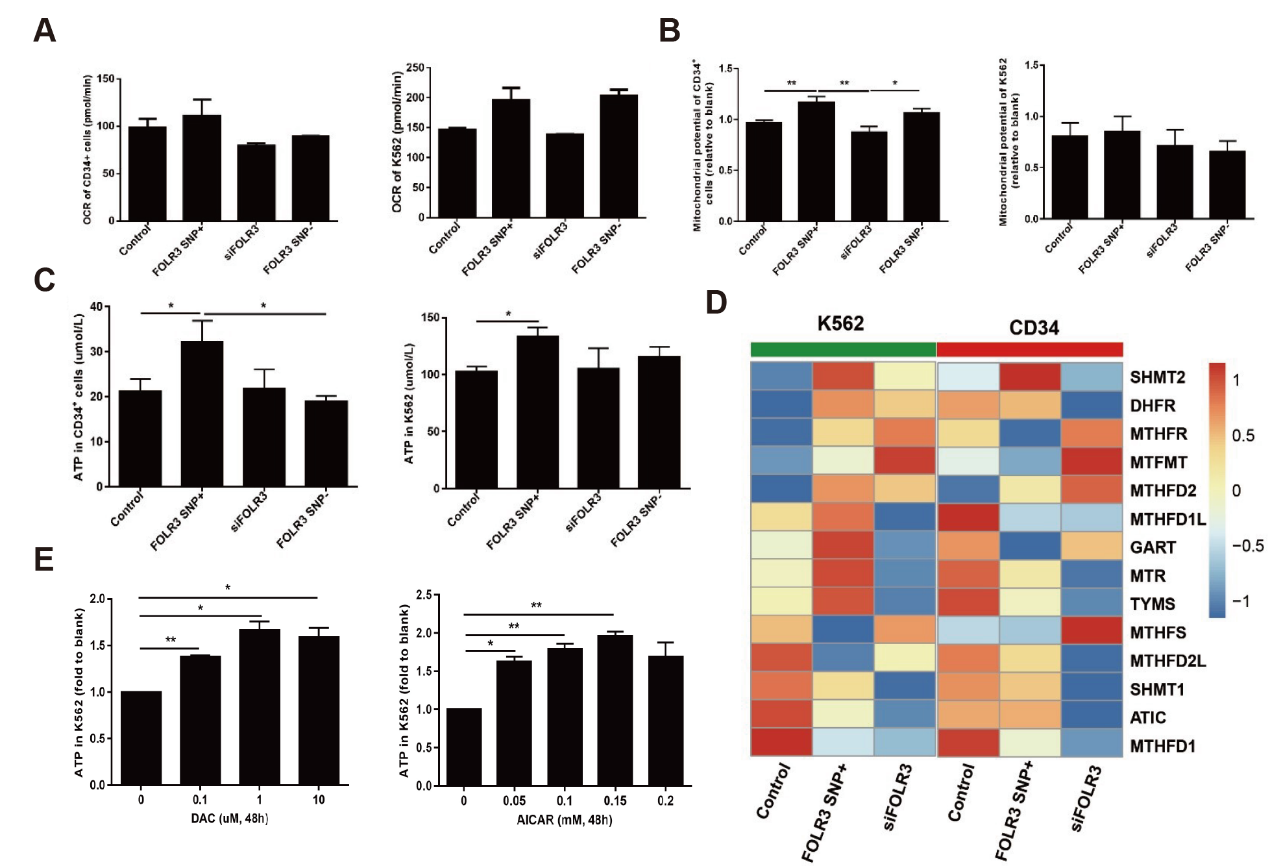


**Supplementary Fig. S5.** FOLR3 SNP enhanced mitochondrial function. **(A)** Basal respirations of CD34+ and K562 cells with different FOLR3 SNP were calculated by Seahorse XF24 analyzer. **(B)** Effects of FOLR3 SNP on mitochondrial membrane potential in CD34+ and K562 cells cultured for 3 days after transfection and then stained with JC-1 dye for flow cytometry. The average fluorescence intensity of red/green ratios relative to blank is shown. **(C)** ATP concentrations in CD34+ and K562 cells (1×10^5^) with different FOLR3 SNP were detected by luciferase assay. **(D)** mRNA-seq analysis of differentially expressed genes in CML CD34+ and K562 cells with different FOLR3 SNP. **(E)** Effects of DAC and AICAR treatment on ATP production in K562 cells. ^*^*P* < 0.05, ^**^*P* < 0.01.

**Supplementary Tables**

**Supplementary Table S1.** Characteristics of 14 patients who discontinued TKI therapy.

| Characteristic | TFR group (n=7) | Relapsed group (n=7) |
| --- | --- | --- |
| Gender |  |  |
| Male, n (%) | 5 (35.71) | 4 (28.57) |
| Female, n (%) | 2 (14.29) | 3 (21.43) |
| CML at diagnosis |  |  |
| Chronic phase (%) | 100 | 100 |
| Median age (years) | 23 (range: 16-52) | 38 (range: 20-68) |
| Karyotype at diagnosis, n (%) |  |  |
| Ph without ACAs | 4 (28.57) | 3 (21.43) |
| Ph with ACAs | 1 (7.14) | 1 (7.14) |
| Unknown | 2 (14.29) | 3 (21.43) |
| Duration of TKI therapy (months) | 92 (range: 54-130) | 84 (range: 59-118) |
| Treatment history |  |  |
| Prior interferon treatment, n (%) | 1 (7.14) | 4 (28.57) |
| 1st line TKI | 5 (35.71) | 7 (50.00) |
| 1st+2nd line TKI | 2 (14.29) | 0 (0.00) |
| FOLR3 SNP |  |  |
| Positive, n (%) | 3 (21.43) | 0 (0.00) |
| Negative, n (%) | 4 (28.57) | 7 (50.00) |

Abbreviations: CML, chronic myeloid leukemia; ACAs, additional chromosome abnormalities; TKI, tyrosine kinase inhibitors; FOLR3 SNP, folate receptor 3 single nucleotide polymorphisms.

**Supplementary Table S2.** Sample information of 7 relapsed CML samples and 7 nonrelapsed CML samples. The NOISeq package was applied to identify differentially expressed genes (DEGs).

| ID | Name | Relapsed OR not | WGC ID | Sample ID |
| --- | --- | --- | --- | --- |
| 1 | ZCJ | Non-relapsed | WGC066798 | N1 |
| 2 | DDZ | Non-relapsed | WGC066806 | N2 |
| 3 | WCH | Non-relapsed | WGC066807 | N3 |
| 4 | LZZ | Non-relapsed | WGC066808 | N4 |
| 5 | CH | Non-relapsed | WGC066810 | N5 |
| 6 | YM | Non-relapsed | WGC066813 | N6 |
| 7 | TJH | Non-relapsed | WGC066816 | N7 |
| 8 | XM | Relapsed | WGC066801 | R1 |
| 9 | WWJ | Relapsed | WGC066804 | R2 |
| 10 | YXP | Relapsed | WGC066805 | R3 |
| 11 | PHS | Relapsed | WGC066809 | R4 |
| 12 | LJF | Relapsed | WGC066811 | R5 |
| 13 | TZF | Relapsed | WGC066812 | R6 |
| 14 | JWG | Relapsed | WGC066814 | R7 |

**Supplementary Table S3.** Samples of the FOLR3 SNP+, SNP- mimics and siFOLR3 for three CML primitive CD34+ groups and one K562 group (1 represents have the sample, 0 represents lack the sample). For the DEGs of siFOLR3 in the CD34+ group (siFOLR3 vs. blank), because only group 1 had an TA insertion in rs139130389 and only the blank sample of group 1 had FOLR3 expressed, the DEGs of siFOLR3 were based on group 1 by FC > 2 or FC < 0.5 and FPKM > 10. For the DEGs of FOLR3 SNP+ and SNP- in the CD34+ groups (FOLR3 SNP+/- vs. blank), we applied FC > 1.25 or FC < 0.8 and FPKM > 10. For the DEGs of the FOLR3 SNP+ in the K562 group (FOLR3 SNP+ vs. blank), we applied FC > 2 or FC < 0.5 and FPKM > 10. The DEGs of control (FC > 2 or FC < 0.5 and FPKM > 10) should be excluded (to remove the blank control effects).

| Type | Group | Blank | | Control | FOLR3 SNP+ | siFOLR3 | FOLR3 SNP- |
| --- | --- | --- | --- | --- | --- | --- | --- |
| CD34 | Group 1 | 1 | 0 | | 1 | 1 | 0 |
|  | Group 2 | 1 | 1 | | 1 | 1 | 1 |
|  | Group 3 | 1 | 1 | | 1 | 1 | 1 |
| K562 | K562 | 1 | 1 | | 1 | 1 | 0 |

**Supplementary Table S4.** Gene list of 5 mitochondrial complexes (source from the gene ontology database), ATP synthesis (source from the gene ontology database), mitochondrial localization proteins (source from the MitoCarta2.0 database) and oxidative phosphorylation (Source from the KEGG database) related proteins.

Mitochondrial Complex1 gene list (GO:0008137)

ACAD9, AIFM1, BCS1L, DMAC1, DMAC2, FOXRED1, NDUFA1, NDUFA10, NDUFA11, NDUFA13, NDUFA2, NDUFA3, NDUFA5, NDUFA8, NDUFA9, NDUFAB1, NDUFAF3, NDUFAF4, NDUFAF5, NDUFAF6, NDUFAF7, NDUFAF8, NDUFB10, NDUFB11, NDUFB2, NDUFB3, NDUFB4, NDUFB5, NDUFB6, NDUFB7, NDUFB8, NDUFB9, NDUFC1, NDUFC2, NDUFS4, NDUFS5, NDUFS7, NDUFS8, NUBPL, OXA1L, TAZ, TIMM21

Mitochondrial Complex2 gene list (GO:0005749)

SDHAF1, SDHAF2, SDHAF3, SDHAF4

Mitochondrial Complex3 gene list (GO:0005750)

CYC1, MT-CO1, PMPCB, UQCC3, UQCR10, UQCRB, UQCRC1, UQCRC2, UQCRFS1, UQCRH, UQCRQ

Mitochondrial Complex4 gene list (GO:0033617)

AA467197, COX4I1, COX4I1, COX4I2, COX4I2, COX5A, COX5A, COX5B, COX6A1, COX6A2, COX7C, COX8A, COX8B, COX8C, MT-CO1, MT-CO1, MT-CO2, NDUFA4, NDUFA4, NDUFA4L2, UQCRC2, UQCRFS1

Mitochondrial Complex5 gene list (GO:0005753)

ATP5A1, ATP5B, ATP5C1, ATP5D, ATP5E, ATP5F1, ATP5G1, ATP5G2, ATP5G3, ATP5H, ATP5J, ATP5J2, ATP5K, ATP5L, ATP5O, ATPIF1, MT-ATP6, MT-ATP8, USMG5

Oxidative phosphorylation pathways gene list (map00190)

MT-ATP8, MT-ND4, MT-ND4L, MT-ND1, MT-ND2, MT-ATP6, MT-ND3, MT-ND6, MT-ND5, RPS18, FTH1, GAPDH, RPL10A, RPL34, RPS14, RPL35A, P4HB, RPS15A, LDHB, TPI1, COX8A, UQCRH, CHCHD2, ATP5J2, ATP5B, SLC25A5, NDUFS5, HSPD1, ATP5A1, DBI, COX6A1, ATP5J, TUFM, TKT, COX6B1, CCT7, SOD1, USMG5, TSPO, PRDX6, HSPA9, C1QBP, SSBP1, GPX4, ATP5C1, GPX1, ATPIF1, CAT, ATP5G2, FKBP8, ATP5H, PARK7, GSTO1, PHB2, VDAC3, PRDX5, NDUFB7, GARS, ACADVL, ATP5O, HSD17B4, COX5B, NCOA4, FDPS, VDAC1, HADHA, PRDX2, UQCRC2, GNG5, GPI, SLC25A3, MDH2, GHITM, HK1, COX6C, ATP5F1, NUCB2, FASN, PPIF, NT5DC2, NDUFV2, TRAP1, STOM, COX7A2, COX4I1, UQCRQ, NDUFV1, COX7C, TCIRG1, HIGD2A, NRD1, TARS, COX5A, AK2, SOD2, MDH1, SHMT2, MRPL12, PRDX3, CYC1, NDUFB9, UQCRC1, UQCR10, GSTK1, PSMA6, BAX, NDUFB8, KARS, NME4, NDUFAB1

ATP synthesis gene list (GO:0042773)

ATP5A1, ATP5B, ATP5C1, ATP5D, ATP5E, ATP5F1, ATP5G1, ATP5G2, ATP5G3, ATP5H, ATP5I, ATP5J2, ATP5J, ATP5L, ATP5O, ATP5SL, MT-ATP6, MT-ATP8

ROS process gene list (GO:0072593)

AKT1, ALOX12, AOX1, ARG2, ATP7A, ATPIF1, BCL2, BNIP3, CAT, CAV1, CCS, CPS1, CTGF, CYB5R4, CYBA, CYBB, CYP1A1, CYP1A2, CYP1B1, CYR61, DDAH2, DDIT4, DHFR, DNM2, DRD5, DUOX1, DUOX2, DUOXA1, DUOXA2, EDN1, EGFR, EPHX2, EPX, FANCC, GBF1, GCH1, GCHFR, GLS2, GPX1, GPX3, GPX4, HBB, HSP90AA1, IL19, LPO, LRRK2, LYPLA1, MAOB, MPO, MPV17, MT-ND2, MT3, NCF1, NCF2, NDUFA13, NDUFAF2, NDUFS1, NDUFS3, NDUFS4, NNT, NOS1, NOS2, NOS3, NOSIP, NOSTRIN, NOX1, NOX3, NOX4, NOX5, NOXA1, NOXO1, NQO1, NRROS, P2RX4, P2RX7, PARK7, PAX2, PDGFB, PDK4, PLA2R1, PMAIP1, POR, PRDX1, PRDX2, PRDX3, PRDX4, PRDX6, PREX1, PRG3, PRKG2, PTS, PXDN, PXDNL, RFK, RORA, SFTPD, SH3PXD2A, SH3PXD2B, SLC7A2, SOD1, SOD2, SOD3, SPR, TLR2, TPO, VAV1, WASL, ZDHHC21

Mitochondrial localization gene list (MitoCart2.0)

CYC1, SDHB, COQ7, SDHA, UQCRC1, COQ5, PDHA1, COQ9, MRPL12, ATP5D, COX5A, ISCA2, PMPCB, UQCRFS1, ATP5A1, OGDH, PDHB, UQCRC2, SDHD, MRPS35, UQCRQ, MRPL53, DBT, PDK4, MDH2, MRPS27, CS, GRPEL1, DLAT, LRPPRC, DLST, PDHX, GFM1, MPC2, NDUFS1, MRPL46, ATP5E, SLC25A3, MRPS23, FH, PMPCA, ATP5B, C6orf57, UQCR10, ISCA1, SUCLA2, COQ3, IARS2, MRPS15, IDH3A, COX11, ETFDH, TIMM10, MRPL34, MRPL2, BCKDHA, UQCRH, HIGD2A, ATP5O, ECHS1, LETMD1, COX6A1, COX15, AFG3L2, HADHA, ETFA, NDUFS7, CPT2, BCKDHB, IDH3B, LARS2, ACADS, LETM1, ATP5I, OPA1, AUH, SUCLG1, NDUFV2, COQ6, MRPL43, ABHD11, ATP5J, NDUFB8, LONP1, DLD, AIFM1, ECHDC3, APOOL, MRPL10, MRRF, NDUFS8, ACADM, IMMT, TIMM9, SLC25A4, SAMM50, NDUFS2, NDUFV1, ACO2, SUPV3L1, FECH, MTIF2, PHB, HIBCH, MRPS2, HSPA9, SURF1, PRDX3, GHITM, GUF1, TIMM13, LYRM4, MRPL16, MRPL40, IDH3G, SDHC, NDUFB5, SDHAF2, COQ10A, C21orf33, TXN2, MRPS18A, COX6C, NDUFB9, MTCH2, NDUFA6, SLC25A20, MRPL1, TIMM44, COX17, MINOS1, SOD2, COX6B1, VDAC1, CLPP, HADH, ACADL, ACAT1, MALSU1, CLPX, NDUFS4, MRPL4, C1QBP, PITRM1, UQCC1, MRPL55, MRM1, MECR, MRPL44, HSDL2, MRPS14, ABCB8, ATPAF2, NDUFA9, COA6, POLRMT, ISCU, RTN4IP1, OGDHL, ATP5C1, BCKDK, GFM2, NDUFAF4, SLC25A35, SLC25A11, TIMM8B, MCAT, IBA57, PHB2, DAP3, CMC2, SDHAF1, MRPS17, ADHFE1, NFS1, NDUFAF5, MRPL17, TACO1, MFN1, PPTC7, MRPL11, COX4I1, NDUFS6, MRPL13, OXCT1, COX5B, PDK2, ALAS1, MRPL33, MTO1, LIAS, NDUFA5, NDUFB6, MTX2, SUCLG2, FDX1, SLC25A1, MRPS28, TIMM50, VDAC2, SLC25A42, TOMM7, ECH1, PET112, BCS1L, ERAL1, CMC1, MRPL28, TSFM, FXN, NFU1, YARS2, MRPL21, ALDH4A1, TOMM40L, NDUFAF1, NDUFB10, ACADSB, MRPS9, ADCK3, ACADVL, COQ4, MRPL15, NDUFA2, MRPL24, HSPD1, NDUFS5, AK3, CYCS, MIPEP, LYRM7, CRAT, PCCB, MRPS7, MRPL3, PRODH, PCCA, MCCC1, MRPS12, CLPB, PDK1, MRPL49, COX7A2, TMEM126A, ECHDC2, HCCS, HIBADH, MRPL19, MRPL36, SLC25A30, BDH1, FARS2, ABCB7, MTX1, NDUFA7, TIMM17A, ALDH9A1, MRPS18C, MARS2, ALDH6A1, FDXR, GATC, TRAP1, ACAD8, ALDH2, PPIF, TIMM22, IVD, L2HGDH, ETHE1, MRPL20, SLC25A5, SLC25A12, MRPS21, TOMM22, ACAA2, MRPL30, DNAJA3, NDUFB2, MRPS34, ETFB, LACE1, ATPIF1, ATP5F1, COX7C, CHCHD3, COQ10B, ACSF3, POLDIP2, SLC25A10, SLC25A13, PDK3, ME3, MRPL22, IDH2, GCDH, MRPL47, PPA2, MRPL9, CHCHD10, WARS2, SLC25A19, CBR4, SMDT1, HAGH, COX7A1, MTG1, COX14, NDUFA12, MRPS16, MTERFD3, LYRM5, GPD2, NDUFA10, NDUFC2, SLIRP, ATP6, COX2, TIMM21, NDUFA8, GLS, NDUFB11, DHTKD1, FAHD2A, HSD17B8, HINT2, MRPS5, C6orf136, SPRYD4, LIPT2, DECR1, MRPS26, SLC25A15, NDUFV3, BPHL, STOML2, MSRB2, LACTB, TOMM40, SLC25A25, ADCK5, ACAD10, VWA8, CCDC90B, DNAJC11, ALKBH7, GLDC, MPC1L, THEM4, SSBP1, MRPL27, HSCB, MRPS10, AK4, ATAD3A, MRPL37, PTGES2, TXNRD2, ACSM5, CISD1, MRPL41, MARC2, MRPS6, MRPL23, SQRDL, SCO1, GCAT, MTHFD1L, ECI2, UQCRB, ATP5H, ATP5L, CCDC58, MCCC2, MCEE, MOCS1, MUT, SLC25A51, SPG7, ATP5G3, ATAD1, OXNAD1, MTG2, DARS2, MRPL51, PC, CLYBL, PNPLA8, MLYCD, PYURF, TIMM17B, MRPL32, SLC25A26, PRODH2, NIT2, FDX1L, ME2, TUFM, COX6A2, SLC30A9, TAMM41, TIMM23, RMND1, MFN2, DNLZ, CHCHD4, NDUFAB1, ACSM1, COA5, COQ2, PDSS2, EHHADH, NDUFS3, ABCB10, CKMT2, MRPS25, ACAD9, COX16, FAM210A, ACOT13, CPT1A, GK, DHRS4, PRELID2, CARS2, GBAS, NDUFB7, C19orf70, GLRX2, MRPS30, SCO2, HSD17B10, NARS2, RPUSD3, SUOX, SARDH, HRSP12, COX8A, SPR, MPV17, ZADH2, MRPL18, ALDH1B1, CHCHD1, TIMM8A, VDAC3, SLC25A24, MPC1, SLC25A22, SLC25A31, SLC25A18, TRNT1, IDE, GSTK1, VARS2, HSPE1, MRPL54, MRPL38, ICT1, MTCH1, TRIAP1, PTCD3, COX10, AKAP1, RNMTL1, AK2, TMEM70, NOA1, MCUR1, TMEM14C, NDUFC1, MRPL35, NDUFA3, PDSS1, SLC25A16, C7orf55, ACSM3, NDUFAF6, UCP1, SFXN1, GLRX5, PTRH1, MRPS11, XPNPEP3, PDP1, ECI1, SLC25A21, SLC25A29, SLC25A45, ACN9, SLMO2, SLC25A39, NDUFB3, REXO2, GATM, TIMM10B, SARS2, CHDH, MRPL57, C8orf82, EARS2, GRHPR, MTFMT, PTCD2, ECHDC1, HARS2, NDUFA4, NDUFA13, APOA1BP, TMEM11, SELO, NAGS, HSD17B4, NDUFAF7, OAT, MRPL50, COX7B, RARS2, OXLD1, LACTB2, NDUFA11, AURKAIP1, CHCHD7, SHMT2, MTHFD2, RMDN1, CPS1, MRPL14, C6orf203, RDH13, TRMT2B, MRPS24, COA3, GSR, ENDOG, BCAT2, TEFM, ACSF2, TRMU, MRPS22, LAP3, GLUD1, HMGCL, FHIT, MRPS33, NUBPL, LYRM2, MPV17L, GADD45GIP1, TFAM, ALDH18A1, OXSM, CCBL2, TARS2, TBRG4, TMEM65, ROMO1, PRDX5, DNAJC15, C2orf47, HIGD1A, C16orf91, RHOT2, SLC25A44, HAO2, OXA1L, SLC25A46, RDH14, C14orf2, CA5B, COASY, CISD3, GSTZ1, COX6B2, GLOD4, ACAA1, CROT, PDF, COX19, AMT, PDPR, NLN, BOLA1, ALAS2, GRPEL2, ALDH7A1, CECR5, GFER, IMMP2L, COX4I2, MRPL39, AADAT, FUNDC2, LYPLA1, PDHA2, MTRF1L, GTPBP3, MTHFD2L, YME1L1, ACOT2, APOPT1, MMAB, PPOX, ABCD3, SIRT3, LDHD, MRPL45, C12orf65, IMMP1L, POLG, ATPAF1, BLOC1S1, ALDH1L2, COX18, CPOX, NNT, C17orf89, XRCC6BP1, DCAKD, ATP5J2, SLC16A1, SFXN3, LONP2, AASS, DIABLO, COX8C, PXMP2, AGXT2, GLS2, APOO, ACAD11, C5orf63, HOGA1, TTC19, NGRN, TCAIM, GCSH, SIRT5, GPT2, TOMM6, FAM162A, ABCB6, MACROD1, TST, COX1, MTFR1L, SDR39U1, DMGDH, SLC25A14, SLMO1, SLC25A17, CHCHD5, TOMM70A, NIT1, FAHD1, MCU, CYP27A1, ND2, ND4, ND5, MDH1, KIAA0141, FASTKD1, MRPS31, ALDH1L1, PTCD1, HADHB, TRIT1, CRLS1, GLYCTK, SLC25A32, AARS2, EXOG, CHCHD2, KMO, SFXN5, ELAC2, GPX1, CPT1B, ME1, FIS1, PRELID1, UCP3, SLC25A27, SLC25A40, SLC25A23, HEMK1, SLC25A36, OMA1, LETM2, DHRS1, STOM, METTL5, MSRA, NUDT8, NMNAT3, SLC25A38, MPV17L2, ACLY, ABCD2, FLAD1, LIPT1, C14orf159, PCK2, NADK2, HK2, MPST, NUDT13, ACACA, ABAT, HMGCS2, ALDH5A1, CKMT1A, PISD, NDUFB4, UQCR11, FAM136A, TOMM5, HTRA2, GPI, NDUFA1, CYTB, GUK1, D2HGDH, OPA3, AKR1B10, PHYH, NDUFAF2, ECSIT, PARL, MRPL42, DNAJC4, PAM16, ALDH3A2, ACSL6, TYSND1, MRS2, ABHD10, HINT3, PXMP4, OSGEPL1, NUDT2, CYB5B, GRSF1, MRPS18B, DNAJC30, CAT, DGUOK, ACOT9, ACSS1, RFK, STARD7, CHPT1, ATP8, TIMMDC1, SPATA19, HTATIP2, AKR7A2, DUT, KIAA0100, COX3, DHRS7B, MTRF1, TMEM143, NT5M, RHOT1, NME4, MTHFS, C19orf52, PNPO, IDH1, MICU2, PCBD2, QRSL1, ADCK1, PRDX6, TKT, NIPSNAP1, GARS, ACSM2A, SLC25A33, DHRSX, LYRM9, RAB24, PARS2, MRPL52, PUS1, GOT2, CARKD, ND3, ACCS, PTRH2, PGS1, OXR1, LYPLAL1, DNAJC28, GSTO1, FPGS, TMEM205, SLC25A53, AIFM3, MGST1, ABCA13, MTFP1, TUBB3, COA4, NIF3L1, PUSL1, SDSL, BAX, MAVS, METTL17, ACACB, ARG2, MTPAP, CCDC51, SUGCT, DNM1L, NRD1, TMEM126B, NSUN4, ACSS3, FAM210B, RBFA, CISD2, METAP1D, AHCYL1, NIPSNAP3A, MARC1, FUNDC1, ACSL1, COX20, HSDL1, CBR3, MTERFD2, NUDT19, HEBP1, PTPMT1, PGAM5, DTYMK, KARS, STAR, DNAJC19, HMBS, OTC, COX7A2L, MTERFD1, SFXN2, FASN, PINK1, TMEM177, TXNRD1, THNSL1, SLC37A4, FDPS, IDI1, TOP3A, FTMT, LDHAL6B, ATXN2, MTFR1, DDAH1, BCL2L13, TRMT10C, ACOT7, TFB1M, ARF5, CCDC109B, C10orf2, MMADHC, ALKBH3, PYCR2, RPS18, TSTD1, RDH11, HSPB7, LYRM1, OSBPL1A, RECQL4, PET100, HK1, GDAP1, MTIF3, UNG, GLYAT, SHMT1, ISOC2, PTS, PPWD1, ATP5G1, SLC16A11, QDPR, SLC16A7, ABCB9, ADCK4, GTPBP10, TARS, TSPO, FTH1, TRMT1, PSMA6, ADCK2, ACOX3, RAB35, TSTD3, MRPS36, PDP2, AGPAT5, FAM195A, UCP2, SLC25A37, SLC25A28, SLC25A48, SLC25A43, SLC25A47, SLC25A41, SLC25A34, PACSIN2, ACO1, MLH1, MTHFD1, EEFSEC, EPHX2, DHCR24, NUDT5, MICU1, SOD1, SERHL2, WDR81, NDUFAF3, BOLA3, OCIAD2, RSAD1, NCEH1, CHCHD6, TPI1, FASTKD2, PEX11B, TMEM186, OBSCN, CYB5A, PIF1, FABP1, ABCD1, GAPDH, PROSC, STOML1, DBI, PI4KA, PNPT1, TMBIM4, TOP1MT, C15orf40, RNASEH1, ABCA9, SCP2, NT5DC3, PTPN4, CMC4, ACOX1, LDHB, YBEY, HINT1, PKLR, MUL1, TK2, AGMAT, CMPK2, BID, ARL2, SYNJ2BP, MFF, DCXR, NUDT9, AMACR, NUDT6, ATP5SL, FTSJ2, BAK1, DHODH, MAOB, MGST3, ACP6, MRPL48, AGXT, CYB5R3, DDX28, BNIP3, MUTYH, GTPBP6, CYP11A1, OCIAD1, METTL8, RPUSD4, NME6, FKBP8, NLRX1, FOXRED1, TFB2M, PPM1K, ATP5G2, ND1, MSRB3, NIPSNAP3B, NTHL1, MARCH5, SFXN4, SLC25A6, OGG1, QTRT1, AIFM2, PRDX2, FBXL4, GPAM, ATP5S, RMDN3, FAM213A, USMG5, PET117, MAOA, C15orf61, IFI27, MMACHC, RPS15A, CRYZ, PYCR1, KRT5, RPL10A, TMLHE, TOMM20, RPS14, COA7, MIEF1, SCCPDH, C15orf48, BNIP3L, FASTKD5, PARK7, RARS, KIF1B, COA1, ND4L, SIRT4, UQCC2, PDE12, NME3, DHX30, FASTK, METTL15, CYP24A1, PANK2, CA5A, NT5DC2, ACYP2, ATAD3B, TRUB2, COMTD1, CPT1C, C3orf33, SND1, ANGEL2, PRDX4, CCT7, P4HB, APEX2, PAICS, MGARP, HDHD3, PSTK, CYP11B2, ATIC, WBSCR16, FAM185A, PMAIP1, TXNDC12, ABCF2, TDRKH, CYP27B1, POLG2, SLC30A6, DUSP26, MTERF, GPX4, SLC22A4, COMT, C12orf10, SPATA20, GNG5, CCDC127, CRY1, ACSL4, MGME1, SEPT4, EMC2, EFHD1, C20orf24, SPTLC2, AGR2, NT5C, NCOA4, NEU4, AGK, DUS2, TOMM34, KIAA0391, TCHP, CKMT1B, NDUFB1, ATP5J2-PTCD1, CASP8, BCL2L1, RPL35A, ARMC10, CDC25C, RAB32, CYB5R2, RAB11FIP5, THG1L, GOLPH3, PREPL, FASTKD3, CEP89, NUCB2, TCIRG1, NBR1, RCN2, FKBP10, SETD9, RPIA, LAMC1, BAD, BCL2, BCL2L2, DMPK, DNA2, ND6, ALKBH1, PICK1, AKAP10, PAK7, NSUN3, PABPC5, PRSS35, ATP10D, SERAC1, STX17, TRMT61B, PLGRKT, RPL34, SECISBP2, MTCP1, C10orf10, C2orf69, ASAH2, CLIC4, SNAP29, TRMT11, ARMS2

**Supplementary Table S5.** Mapping result of the sanger sequencing of 112 CML samples. We detected 10 out of 112 had TA insertion.

| 100_FOLR3-F_TSS20180 TGGTAAGATGGAACCCACCTGCAAACGCCACTTTATCCAGGACAGCTGTCTC--TGAGTG |
| --- |
| 101_FOLR3-F_TSS20180 TGGTAAGATGGAACCCACCTGCAAACGCCACTTTATCCAGGACAGCTGTCTC--TGAGTG |
| 102_FOLR3-F_TSS20180 TGGTAAAATGGAACCCACCTGCAAACGCCACTTTATCCATGACAGCTGTCTC--TGAGTG |
| 104_FOLR3-F_TSS20180 TGGTAAGATGGAACCCACCTGCAAGCGCCACTTTATCCAGGACAGCTGTCTC--TGAGTG |
| 105_FOLR3-F_TSS20180 TGGTAAGATGGAACCCACCTGCAAGCGCCACTTTATCCAGGACAGCTGTCTC--TGAGTG |
| 106_FOLR3-F_TSS20180 TGGTAAGATGGAACCCACCTGCAAACGCCACTTTATCCAGGACAGCTGTCTC--TGAGTG |
| 107_FOLR3-F_TSS20180 TGGTAAGATGGAACCCACCTGCAAACGCCACTTTATCCAGGACAGCTGTCTC--TGAGTG |
| 109_FOLR3-F_TSS20180 TGGTAAGATGGAACCCACCTGCAAACGCCACTTTATCCAGGACAGCTGTCTC--TGAGTG |
| 110_FOLR3-F_TSS20180 TGGTAAGATGGAACCCACCTGCAAACGCCACTTTATCCAGGACAGCTGTCTC--TGAGTG |
| 111_FOLR3-F_TSS20180 TGGTAAGATGGAACCCACCTGCAAGCGCCACTTTATCCAGGACAGCTGTCTC--TGAGTG |
| 112_FOLR3-F_TSS20180 TGGTAAGATGGAACCCACCTGCAAGCGCCACTTTATCCAGGACAGCTGTCTC--TGAGTG |
| 113_FOLR3-F_TSS20180 TGGTAAGATGGAACCCACCTGCAAACGCCACTTTATCCAGGACAGCTGTCTC--TGAGTG |
| 114_FOLR3-F_TSS20180 TGGTAAGATGGAACCCACCTGCAAGCGCCACTTTATCCAGGACAGCTGTCTC--TGAGTG |
| 115_FOLR3-F_TSS20180 TGGTAAGATGGAACCCACCTGCAAGCGCCACTTTATCCAGGACAGCTGTCTC--TGAGTG |
| 116_FOLR3-F_TSS20180 TGGTAAGATGGAACCCACCTGCAAGCGCCACTTTATCCAGGACAGCTGTCTC--TGAGTG |
| 117_FOLR3-F_TSS20180 TGGTAAGATGGAACCCACCTGCAAGCGCCACTTTATCCAGGACAGCTGTCTCTATGAGTG |
| 118_FOLR3-F_TSS20180 TGGTAAGATGGAACCCACCTGCAAGCGCCACTTTATCCAGGACAGCTGTCTC--TGAGTG |
| 119_FOLR3-F_TSS20180 TGGTAAGATGGAACCCACCTGCAAACGCCACTTTATCCAGGACAGCTGTCTC--TGAGTG |
| 122_FOLR3-F_TSS20180 TGGTAAGATGGAACCCACCTGCAAGCGCCACTTTATCCAGGACAGCTGTCTC--TGAGTG |
| 124_FOLR3-F_TSS20180 TGGTAAGATGGAACCCACCTGCAAGCGCCACTTTATCCAGGACAGCTGTCTC--TGAGTG |
| 125_FOLR3-F_TSS20180 TGGTAAGATGGAACCCACCTGCAAACGCCACTTTATCCAGGACAGCTGTCTC--TGAGTG |
| 126_FOLR3-F_TSS20180 TGGTAAGATGGAACCCACCTGCAAGCGCCACTTTATCCAGGACAGCTGTCTC--TGAGTG |
| 128_FOLR3-F_TSS20180 TGGTAAGATGGAACCCACCTGCAAACGCCACTTTATCCAGGACAGCTGTCTC--TGAGTG |
| 130_FOLR3-F_TSS20180 TGGTAAGATGGAACCCACCTGCAAGCGCCACTTTATCCAGGACAGCTGTCTC--TGAGTG |
| 133_FOLR3-F_TSS20180 TGGTAAGATGGAACCCACCTGCAAGCGCCACTTTATCCAGGACAGCTGTCTC--TGAGTG |
| 134_FOLR3-F_TSS20180 TGGTAAGATGGAACCCACCTGCAAGCGCCACTTTATCCAGGACAGCTGTCTC--TGAGTG |
| 135_FOLR3-F_TSS20180 TGGTAAGATGGAACCCACCTGCAAACGCCACTTTATCCAGGACAGCTGTCTC--TGAGTG |
| 136_FOLR3-F_TSS20180 TGGTAAGATGGAACCCACCTGCAAGCGCCACTTTATCCAGGACAGCTGTCTC--TGAGTG |
| 137_FOLR3-F_TSS20180 TGGTAAGATGGAACCCACCTGCAAGCGCCACTTTATCCAGGACAGCTGTCTC--TGAGTG |
| 138_FOLR3-F_TSS20180 TGGTAAGATGGAACCCACCTGCAAGCGCCACTTTATCCAGGACAGCTGTCTC--TGAGTG |
| 140_FOLR3-F_TSS20180 TGGTAAGATGGAACCCACCTGCAAGCGCCACTTTATCCAGGACAGCTGTCTC--TGAGTG |
| 141_FOLR3-F_TSS20180 TGGTAAGATGGAACCCACCTGCAAGCGCCACTTTATCCAGGACAGCTGTCTC--TGAGTG |
| 143_FOLR3-F_TSS20180 TGGTAAGATGGAACCCACCTGCAAGCGCCACTTTATCCAGGACAGCTGTCTC--TGAGTG |
| 145_FOLR3-F_TSS20180 TGGTAAGATGGAACCCACCTGCAAGCGCCACTTTATCCAGGACAGCTGTCTCTATGAGTG |
| 146_FOLR3-F_TSS20180 TGGTAAGATGGAACCCACCTGCAAGCGCCACTTTATCCAGGACAGCTGTCTC--TGAGTG |
| 147_FOLR3-F_TSS20180 TGGTAAGATGGAACCCACCTGCAAGCGCCACTTTATCCAGGACAGCTGTCTC--TGAGTG |
| 148_FOLR3-F_TSS20180 TGGTAAGATGGAACCCACCTGCAAGCGCCACTTTATCCAGGACAGCTGTCTC--TGAGTG |
| 149_FOLR3-F_TSS20180 TGGTAAGATGGAACCCACCTGCAAGCGCCACTTTATCCAGGACAGCTGTCTC--TGAGTG |
| 150_FOLR3-F_TSS20180 TGGTAAGATGGAACCCACCTGCAAGCGCCACTTTATCCAGGACAGCTGTCTC--TGAGTG |
| 18_FOLR3-F_TSS201802 TGGTAAAATGGAACCCACCTGCAAACGCCACTTTATCCAGGACAGCTGTCTC--TGAGTG |
| 22_FOLR3-F_TSS201707 TGGTAAGATGGAACCCACCTGCAAGCGCCACTTTATCCAGGACAGCTGTCTCTATGAGTG |
| 26_FOLR3-F_TSS201802 TGGTAAGATGGAACCCACCTGCAAGCGCCACTTTATCCAGGACAGCTGTCTC--TGAGTG |
| 27_FOLR3-F_TSS201802 TGGTAAAATGGAACCCACCTGCAAACGCCATTTTATCCAGGACCCTTGTCTC--TGAGTG |
| 28_FOLR3-F_TSS201802 TGGTAAAATGGAACCCACCTGCAAACGCCACTTTATCCAGGACAGCTGTCTC--TGAGTG |
| 29_FOLR3-F_TSS201802 TGGTAAAATGGAACCCACCTGCAAACGCCACTTTATCCAGGACAGCTGTCTC--TGAGTG |
| 30_FOLR3-F_TSS201802 TGGTAAAATGGAACCCACCTGCAAGCGCCACTTTATCCAGGACAGCTGTCTC--TGAGTG |
| 32_FOLR3-F_TSS201802 TGGTAAAATGGAACCCACCTGCAAACGCCACTTTATCCAGGACAGCTGTCTC--TGAGTG |
| 34_FOLR3-F_TSS201802 TGGTAAAATGGAACCCACCTGCAAACGCCACTTTATCCAGGACAGCTGTCTC--TGAGTG |
| 35_FOLR3-F_TSS201802 TGGTAAGATGGAACCCACCTGCAAACGCCACTTTATCCAGGACAGCTGTCTC--TGAGTG |
| 36_FOLR3-F_TSS201802 TGGTAAGATGGAACCCACCTGCAAGCGCCACTTTATCCAGGACAGCTGTCTC--TGAGTG |
| 37_FOLR3-F_TSS201802 TGGTAAAATGGAACCCACCTGCAAGCGCCACTTTATCCAGGACAGCTGTCTC--TGAGTG |
| 38_FOLR3-F_TSS201802 TGGTAAAATGGAACCCACCTGCAAACGCCACTTTATCCAGGACAGCTGTCTC--TGAGTG |
| 39_FOLR3-F_TSS201802 TGGTAAGATGGAACCCACCTGCAAGCGCCACTTTATCCAGGACAGCTGTCTC--TGAGTG |
| 3_FOLR3-F_TSS2017071 TGGTAAAATGGAACCCACCTGCAAGCGCCACTTTATCCAGGACAGCTGTCTC--TGAGTG |
| 40_FOLR3-F_TSS201802 TGGTAAGATGGAACCCACCTGCAAGCGCCACTTTATCCAGGACAGCTGTCTC--TGAGTG |
| 41_FOLR3-F_TSS201802 TGGTAAGATGGAACCCACCTGCAAGCGCCACTTTATCCAGGACAGCTGTCTC--TGAGTG |
| 42_FOLR3-F_TSS201802 TGGTAAGATGGAACCCACCTGCAAGCGCCACTTTATCCAGGACAGCTGTCTC--TGAGTG |
| 43_FOLR3-F_TSS201802 TGGTAAGATGGAACCCACCTGCAAGCGCCACTTTATCCAGGACAGCTGTCTC--TGAGTG |
| 44_FOLR3-F_TSS201802 TGGTAAGATGGAACCCACCTGCAAGCGCCACTTTATCCAGGACAGCTGTCTC--TGAGTG |
| 45_FOLR3-F_TSS201802 TGGTAAGATGGAACCCACCTGCAAGCGCCACTTTATCCAGGACAGCTGTCTC--TGAGTG |
| 46_FOLR3-F_TSS201802 TGGTAAAATGGAACCCACCTGCAAACGCCACTTTATCCAGGACAGCTGTCTC--TGAGTG |
| 49_FOLR3-F_TSS201802 TGGTAAAATGGAACCCACCTGCAAACGCCATTTTATCCAGGACAGTTGTCTC--TGAGTG |
| 50_FOLR3-F_TSS201802 TGGTAAGATGGAACCCACCTGCAAGCGCCACTTTATCCAGGACAGCTGTCTC--TGAGTG |
| 51_FOLR3-F_TSS201802 TGGTAAAATGGAACCCACCTGCAAACGCCATTTTATCCAGGACAGTTGTCTC--TGAGTG |
| 52_FOLR3-F_TSS201802 TGGTAAAATGGAACCCACCTGCAAACGCCACTTTATCCAGGACAGCTGTCTC--TGAGTG |
| 53_FOLR3-F_TSS201802 TGGTAAAATGGAACCCACCTGCAAGCGCCACTTTATCCAGGACAGCTGTCTC--TGAGAG |
| 54_FOLR3-F_TSS201802 TGGTAAAATGGAACCCACCTGCAAACGCCATTTTATCCAGGACACTTGTCTC--TGAGTG |
| 55_FOLR3-F_TSS201802 TGGTAAGATGGAACCCACCTGCAAGCGCCACTTTATCCAGGACAGCTGTCTC--TGAGTG |
| 56_FOLR3-F_TSS201802 TGGTAAAATGGAACCCACCTGCAAACGCCACTTTATCCAGGACACTTGTCTC--TGAGTG |
| 57_FOLR3-F_TSS201802 TGGTAAAATGGAACCCACCTGCAAACGCCACTTTATCCAGGACAGCTGTCTC--TGAGTG |
| 58_FOLR3-F_TSS201802 TGGTAAAATGGAACCCACCTGCAAACGCCACTTTATCCAGGACAGCTGTCTC--TGAGTG |
| 59_FOLR3-F_TSS201802 TGGTAAGATGGAACCCACCTGCAAGCGCCACTTTATCCAGGACAGCTGTCTC--TGAGTG |
| 5_FOLR3-F_TSS2017071 TGGTAAGATGGAACCCACCTGCAAGCGCCACTTTATCCAGGACAGCTGTCTCTATGAGTG |
| 60_FOLR3-F_TSS201802 TGGTAAAATGGAACCCACCTGCAAGCGCCACTTTATCCAGGACAGCTGTCTC--TGAGTG |
| 61KONG_FOLR3-F_TSS20 TGGTAAGATGGAACCCACCTGCAAGCGCCACTTTATCCAGGACAGCTGTCTCTATGAGTG |
| 62_FOLR3-F_TSS201802 TGGTAAGATGGAACCCACCTGCAAGCGCCACTTTATCCAGGACAGCTGTCTC--TGAGTG |
| 63_FOLR3-F_TSS201802 TGGTAAGATGGAACCCACCTGCAAGCGCCACTTTATCCAGGACAGCTGTCTC--TGAGTG |
| 64_FOLR3-F_TSS201802 TGGTAAGATGGAACCCACCTGCAAGCGCCACTTTATCCAGGACAGCTGTCTCTATGAGTG |
| 65_FOLR3-F_TSS201802 TGGTAAGATGGAACCCACCTGCAAGCGCCACTTTATCCAGGACAGCTGTCTC--TGAGTG |
| 66_FOLR3-F_TSS201802 TGGTAAAATGGAACCCACCTGCAAACGCCACTTTATCCAGGACAGCTGTCTC--TGAGTG |
| 68_FOLR3-F_TSS201802 TGGTAAAATGGAACCCACCTGCAAGCGCCACTTTATCCAGGACAGCTGTCTC--TGAGTG |
| 69_FOLR3-F_TSS201802 TGGTAAGATGGAACCCACCTGCAAGCGCCACTTTATCCAGGACAGCTGTCTC--TAAGAG |
| 6_FOLR3-F_TSS2017071 TGGTAAAATGGAACCCACCTGCAAGCGCCACTTTATCCAGGACAGCTGTCTC--TGAGTG |
| 70_FOLR3-F_TSS201802 TGGTAAGATGGAACCCACCTGCAAGCGCCACTTTATCCAGGACAGCTGTCTC--TGAGTG |
| 71_FOLR3-F_TSS201802 TGGTAAGATGGAACCCACCTGCAAGCGCCACTTTATCCAGGACAGCTGTCTC--TGAGTG |
| 72_FOLR3-F_TSS201802 TGGTAAGATGGAACCCACCTGCAAGCGCCACTTTATCCAGGACAGCTGTCTC--TGAGTG |
| 74_FOLR3-F_TSS201802 TGGTAAGATGGAACCCACCTGCAAGCGCCACTTTATCCAGGACAGCTGTCTC--TGAGTG |
| 75_FOLR3-F_TSS201802 TGGTAAGATGGAACCCACCTGCAAGCGCCACTTTATCCAGGACAGCTGTCTC--TGAGTG |
| 76_FOLR3-F_TSS201802 TGGTAAAATGGAACCCACCTGCAAACGCCACTTTATCCAGGACAGCTGTCTC--TGAGTG |
| 77_FOLR3-F_TSS201802 TGGTAAGATGGAACCCACCTGCAAGCGCCACTTTATCCAGGACAGCTGTCTC--TGAGTG |
| 78_FOLR3-F_TSS201802 TGGTAAGATGGAACCCACCTGCAAACGCCACTTTATCCAGGACAGCTGTCTC--TGAGTG |
| 79_FOLR3-F_TSS201802 TGGTAAGATGGAACCCACCTGCAAGCGCCACTTTATCCAGGACAGCTGTCTC--TGAGTG |
| 80_FOLR3-F_TSS201802 TGGTAAGATGGAACCCACCTGCAAACGCCACTTTATCCAGGACAGCTGTCTC--TGAGTG |
| 81_FOLR3-F_TSS201802 TGGTAAGATGGAACCCACCTGCAAGCGCCACTTTATCCAGGACAGCTGTCTCTATGAGTG |
| 82_FOLR3-F_TSS201802 TGGTAAAATGGAACCCACCTGCAAACGCCACTTTATCCAGGACAGCTGTCTC--TGAGTG |
| 83_FOLR3-F_TSS201802 TGGTAAGATGGAACCCACCTGCAAGCGCCACTTTATCCAGGACAGCTGTCTCTATGAGTG |
| 84_FOLR3-F_TSS201802 TGGTAAGATGGAACCCACCTGCAAGCGCCACTTTATCCAGGACAGCTGTCTC--TGAGTG |
| 85_FOLR3-F_TSS201802 TGGTAAGATGGAACCCACCTGCAAGCGCCACTTTATCCAGGACAGCTGTCTC--TGAGTG |
| 85MO_FOLR3-F_TSS2018 TGGTAAGATGGAACCCACCTGCAAGCGCCACTTTATCCAGGACAGCTGTCTC--TGAGTG |
| 86_FOLR3-F_TSS201802 TGGTAAGATGGAACCCACCTGCAAGCGCCACTTTATCCAGGACAGCTGTCTC--TGAGTG |
| 87_FOLR3-F_TSS201802 TGGTAAGATGGAACCCACCTGCAAGCGCCACTTTATCCAGGACAGCTGTCTC--TGAGTG |
| 88_FOLR3-F_TSS201802 TGGTAAAATGGAACCCACCTGCAAACGCCATTTTATCCAGGACCCTTGTCTC--TGAGTG |
| 89_FOLR3-F_TSS201802 TGGTAAAATGGAACCCACCTGCAAACGCCATTTTATCCAGGACCCTTGTCTC--TGAGTG |
| 90_FOLR3-F_TSS201802 TGGTAAGATGGAACCCACCTGCAAGCGCCACTTTATCCAGGACAGCTGTCTC--TGAGTG |
| 91_FOLR3-F_TSS201802 TGGTAAGATGGAACCCACCTGCAAACGCCACTTTATCCAGGACAGCTGTCTC--TGAGTG |
| 92_FOLR3-F_TSS201802 TGGTAAGATGGAACCCACCTGCAAGCGCCACTTTATCCAGGACAGCTGTCTC--TGAGTG |
| 93_FOLR3-F_TSS201802 TGGTAAGATGGAACCCACCTGCAAGCGCCACTTTATCCAGGACAGCTGTCTCTATGAGTG |
| 94_FOLR3-F_TSS201802 TGGTAAGATGGAACCCACCTGCAAGCGCCACTTTATCCAGGACAGCTGTCTCTATGAGTG |
| 95_FOLR3-F_TSS201802 TGGTAAGATGGAACCCACCTGCAAGCGCCACTTTATCCAGGACAGCTGTCTC--TGAGTG |
| 96_FOLR3-F_TSS201802 TGGTAAGATGGAACCCACCTGCAAGCGCCACTTTATCCAGGACAGCTGTCTC--TGAGTG |
| 97_FOLR3-F_TSS201802 TGGTAAGATGGAACCCACCTGCAAGCGCCACTTTATCCAGGACAGCTGTCTC--TGAGTG |
| 98_FOLR3-F_TSS2017071 TGGTAAAATGGAACCCACCTGCAAGCGCCACTTTATCCAGGACAGCTGTCTC--TGAGTG |
| Reference-FOLR3 TGGTAAGATGGAACCCACCTGCAAGCGCCACTTTATCCAGGACAGCTGTCTCTATGAGTG |

**Supplementary Table S6.** Relapse rate of FOLR3 SNP+ and FOLR3 SNP- of 87 patients who discontinued TKI therapy.

| Characteristic | Relapse (%) | Non-relapsed (%) |
| --- | --- | --- |
| FOLR3 SNP+ | 2 (13.33) | 13 (86.67) |
| FOLR3 SNP- | 30 (41.67) | 42 (58.33) |

Abbreviations: TKI, tyrosine kinase inhibitor; FOLR3 SNP, folate receptor 3 single nucleotide polymorphisms.
